# The Fungal Histone Acetyl Transferase Gcn5 Controls Virulence of the Human Pathogen *Candida albicans* through Multiple Pathways

**DOI:** 10.1038/s41598-019-45817-5

**Published:** 2019-07-01

**Authors:** Raju Shivarathri, Michael Tscherner, Florian Zwolanek, Nitesh Kumar Singh, Neeraj Chauhan, Karl Kuchler

**Affiliations:** 1 Medical University of Vienna, Max Perutz Labs Vienna, Campus Vienna Biocenter, A-1030 Vienna, Austria; 2 Qia ULB-Epigénétique du Cancer, Faculté Médecine Route de Lennik, 808 Bruxelles, Belgium; 3 Public Health Research Institute, New Jersey Medical School, Rutgers The State University of New Jersey, Newark, NJ 07103 USA; 4 Department of Microbiology, Biochemistry and Molecular Genetics, New Jersey Medical School, Rutgers The State University of New Jersey, Newark, NJ 07103 USA

**Keywords:** Fungal pathogenesis, Transcriptomics

## Abstract

Fungal virulence is regulated by a tight interplay of transcriptional control and chromatin remodelling. Despite compelling evidence that lysine acetylation modulates virulence of pathogenic fungi such as *Candida albicans*, the underlying mechanisms have remained largely unexplored. We report here that Gcn5, a paradigm lysyl-acetyl transferase (KAT) modifying both histone and non-histone targets, controls fungal morphogenesis – a key virulence factor of *C*. *albicans*. Our data show that genetic removal of *GCN5* abrogates fungal virulence in mice, suggesting strongly diminished fungal fitness *in vivo*. This may at least in part arise from increased susceptibility to killing by macrophages, as well as by other phagocytes such as neutrophils or monocytes. Loss of *GCN5* also causes hypersensitivity to the fungicidal drug caspofungin. Caspofungin hypersusceptibility requires the master regulator Efg1, working in concert with Gcn5. Moreover, Gcn5 regulates multiple independent pathways, including adhesion, cell wall-mediated MAP kinase signaling, hypersensitivity to host-derived oxidative stress, and regulation of the Fks1 glucan synthase, all of which play critical roles in virulence and antifungal susceptibility. Hence, Gcn5 regulates fungal virulence through multiple mechanisms, suggesting that specific inhibition of Gcn5 could offer new therapeutic strategies to combat invasive fungal infections.

## Introduction

Invasive fungal infections claim about 1.5 million lives each year^1^. *Candida* species (spp) rank among the top three for four causes of nosocomial infectious diseases^2,3^. While *Candida* spp are typically normal commensal colonizers of mucosal barriers in healthy individuals^4,5^, they can cause life-threatening invasive infections in intensive care unit patients, as well as those with impaired immune defence such as particular neutropenia^6–8^. Invasive candidemia is associated with high mortalities of 35–55%^7,9^ and accounts for up to 10% of nosocomial blood stream infections (BSIs)^10^. Limited therapeutic options to treat invasive fungal infections, and increased emergence of antifungal drug resistance in related species such as *Candida glabrata* and *Candida auris*^11–13^ pose a significant and growing healthcare problem^14,15^.

Pathogenicity mechanisms of *C*. *albicans* include adherence as biofilms, morphogenetic switching, tissue tropism, secretion of hydrolases and metabolic adaptation as well as chromatin remodelling^16–18^. For example, the fungal cell wall, a prime antifungal target^19,20^, is home to many adhesins and undergoes dynamic remodelling during host stress or immune response to evade detection^21–23^. Moreover, three major MAPK signaling pathways, the Mkc1-mediated cell integrity pathway, the Hog1-dependent high osmolarity pathway and the Cek1-mediated invasion and filamentation pathway, respond to environmental stimuli and thus cooperate in regulating *C*. *albicans* virulence^24–28^. Signaling pathways converge at dedicated downstream transcriptional regulators such as Efg1 and Cph1 and others that control signaling integration to regulate morphogenesis, virulence but also immune evasion^29–32^.

Interestingly, most if not all fungal virulence traits are tightly controlled by a dual-layer network that engages transcriptional regulatory networks, whose activity is modulated by specific histone modification enzymes that alter chromatin states. For example, genetic ablation of lysine acetyltransferases and lysine deacetylases (KATs/KDACs) Set3C, Rpd3, Rp31, Hat1, Hst3 and Rtt109 abolishes fungal virulence^33–39^. Indeed, KATs and KDACs cooperate with transcriptional regulators in the control of fungal virulence^18,40,41^ but the molecular mechanisms underlying KATs/KDACs function in fungal pathogenesis remain poorly understood. However, several fungal-specific lysine modifications indicate a potential as valuable therapeutic targets with minimal toxic side effects^40,42,43^.

As a hallmark fungal lysyl acetyltransferase, Gcn5 (general control nonderepressible-5), is a paradigm KAT and member of the evolutionary conserved Gcn5-related N-acetyltransferase family (GNATs). Yeast Gcn5 is part of large transcriptional multiprotein complexes, including SAGA (Spt-Ada-Gcn5 acetyltransferase), ADA (Ada2-Gcn5-Ada3), HAT-A2 and SLIK (SAGA-like). These evolutionary conserved regulatory complexes recruit the basal transcription machinery and coactivators to specific promoters, control chromatin modification and nucleosome remodelling, as well as retrograde signaling^44–47^. For example, Gcn5 is essential for stress response both in fission yeast *Schizosaccharomyces pombe* and budding yeast *Saccharomyces cerevisiae*^48–50^. Further, the UmGcn5 homologue in the maize pathogen *Ustilago maydis* participates in the epigenetic regulation of morphogenesis and pathogenesis^51,52^. The *Aspergillus nidulans* GcnE homologue is crucial for inducing genes responsible for conidiation and conidiophore development^53^. Interestingly, CnGcn5 in *Cryptococcus neoformans*, the primary cause of meningoencephalitis with highest frequencies in HIV patients, controls stress responses and attenuates virulence^54^. Gcn5-mediated histone H3 acetylation is required for conidiation, dimorphic transition and virulence of entomopathogenic fungi *Beauveria bassiana*^55^. *Candida albicans* Gcn5 also attenuates pathogenicity and affects morphogenesis^56^, but the mechanisms of Gcn5-mediated gene regulation, and more importantly, how Gcn5 controls fungal pathogenicity remains largely unknown. Here, we show that Gcn5 controls *C*. *albicans* invasive infections by acting downstream of multiple signaling pathways that control cell wall architecture and surface remodeling. Importantly, Gcn5 critically determines susceptibility to killing by innate immune cells, as well as to the fungicidal action by caspofungin. The data establish Gcn5 as drug target which may be suitable for interfering with invasive fungal infections.

## Results

### Genetic ablation of the Gcn5 histone acetyltransferase impairs filamentation

**Figure 1:**
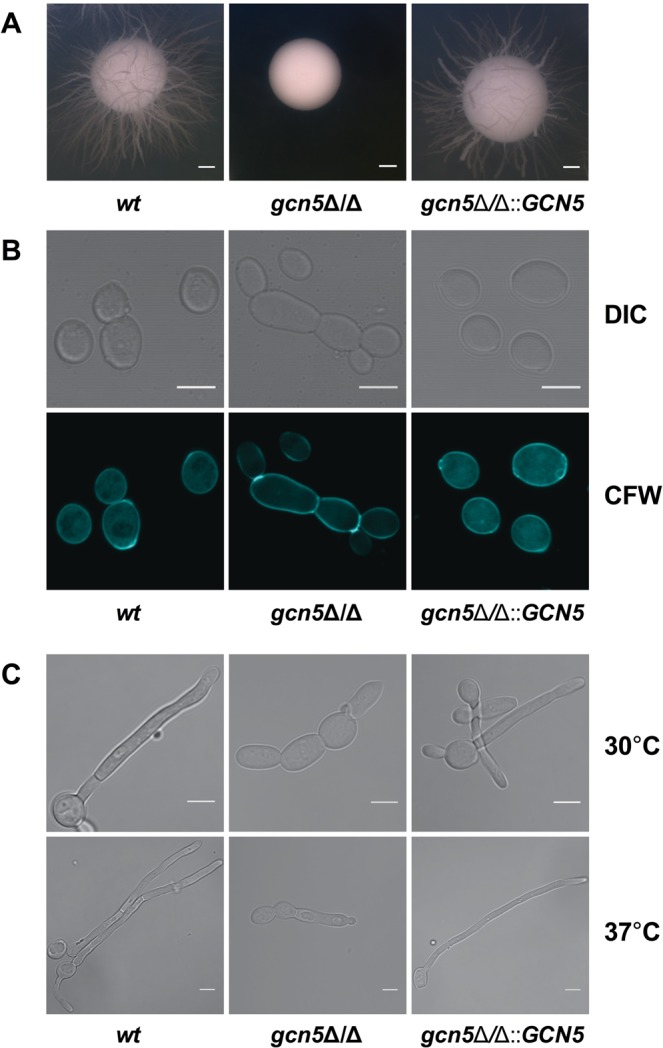
Lack of *GCN5* impairs bud separation, hyphae formation and agar invasion. (**A**) Logarithmically growing cells of SC5314 wild-type (wt), homozygous deletion (*gcn5*Δ/Δ) and restored (*gcn5*Δ/Δ::*GCN5*) Candida strains were plated on YPD plates supplemented with 10% fetal calf serum. Colony morphology was analysed after incubating for 3 days at 37 °C. Photographs were taken using a Discovery V12 Stereoscope equipped with an Axiocam MR5 camera (Zeiss). Scale bar = 1 mm. (**B**) Representative confocal microscopy image showing fungal cells stained with calcofluor white (CFW) to detect cell wall chitin. Cells were fixed in 4% *p*-formaldehyde for 2 hours, washed and stained with CFW 1 mg/ml for 5 min. Differential Interference Contrast (DIC) and UV light images (UV) of the same cells are shown at 60x magnification. Scale bar = 5 µM. (**C**). Representative confocal DIC images showing the hyphal morphology of strains at indicated temperatures. Logarithmically growing cells in YPD supplemented with 10% FCS were fixed in 4% *p*-formaldehyde for 2 hours and washed and images were taken with an LSM 700 Zeiss Confocal microscope at 60x magnification. Scale bar = 5 µM.

First, we asked if the type A KAT, Gcn5, is involved in fungal morphogenesis. We created homozygous deletion (*gcn5*∆/∆) mutants using the recyclable *NAT1* flipper method^57^ in a SC5314 wild type (*wt*) strain background, including a restored *gcn5*∆/∆::*GCN5* strain. While this work was in progress, Chang *et al*. reported that Gcn5 is required for the invasive growth, hyphal elongation in hyphal-inducing conditions^56^. Consistent with this notion, we found that lack of Gcn5 led to a smooth colony morphology on filament-inducing media, indicating a severe morphogenesis defect when compared to wild type control and the reconstituted *gcn5*∆/∆::*GCN5* strain (Fig. 1A). Mutant cells also displayed a morphology defect as indicated by the pseudo-hyphal morphology on complete YPD medium, and by the aberrant chitin deposition as visualized by calcofluor white (CFW) staining (Fig. 1B). In full agreement with a previous report^56^, we show that filamentation of *gcn5*∆/∆ cells was severely impaired, since true hyphae were absent. Moreover, daughter cells often remained attached to mother cells, indicating a separation defect (Fig. 1C).

Since morphological defects often associate with altered drug susceptibilities, we also tested cell growth on solid and liquid media supplemented with various stress agents including caffeine, the cell wall stressor SDS and Congo Red, and specific antifungal agents, as well as different carbon sources (Supplementary Fig. S1). Comprehensive phenotyping showed an altered sensitivity to several stress conditions, including increased susceptibility to antifungal agent caspofungin (CSP), albeit azole sensitivity was not significantly altered (Supplementary Fig. S1A,B). The *gcn5*∆/∆ mutant showed slightly increased resistance to peroxide stress (Supplementary Fig. S1B). Cells lacking *GCN5* showed growth defects in media containing citric acid, ethanol and sodium acetate used as sole carbon sources (Supplementary Fig. S1C,D), but consumed glucose and glycerol like wild type cells. The elevated sensitivity to CSP and SDS was also confirmed by MIC_50_ assays in liquid media, showing that SDS and CSP sensitivities were 12 and ~3-fold elevated in *gcn5*∆/∆, respectively, while caffeine sensitivity was essentially unchanged (Supplementary Fig. S1E). Taken together, these data suggest that Gcn5 is required for normal morphogenesis and cell separation, stress adaptation, carbon source utilization, as well as antifungal susceptibility and cell wall homeostasis.

### Lack of Gcn5 differentially regulates MAPK signaling

**Figure 2:**
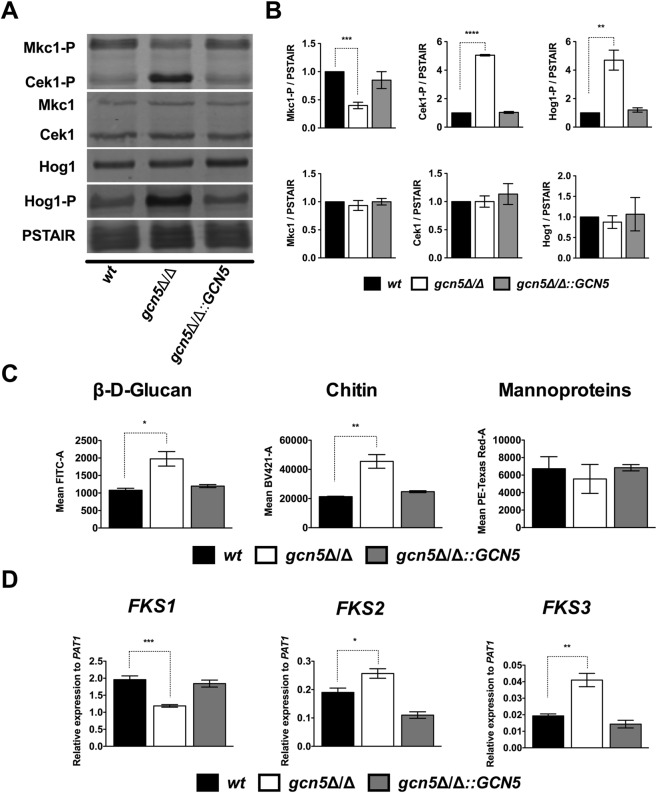
Lack of Gcn5 differentially regulates MAPK signaling and cell wall components. (**A**) Logarithmically growing cultures of SC5314 wild-type (wt), homozygous deletion (*gcn5*Δ/Δ) and restored (*gcn5*Δ/Δ::*GCN5*) Candida strains were used to prepare cell free extracts using the TCA protocol as described in materials and methods. Extracts corresponding to 1 OD_600_ (1 × 10^7^ cells) were fractionated by SDS-Page and blotted for proteins as indicated. Signals from the same whole cell extracts were detected using antibodies for total and phosphorylated MAP kinases. The commercial antibodies recognized Mkc1 and Cek1 (p44/42 MAPK Erk1/2, Cell Signaling), and Hog1 (y-215, Santa Cruz), and phosphorylated Mkc1-P and Cek1-P (Phospho-p44/42 MAPK (Erk1/2), Cell Signaling) and Hog1-P (Phospho-p38, Cell Signaling). Reprobing with PSTAIR antibody (Sigma) recognizing Cdc28 served as a loading control. (**B**) Densitometry analysis was done by using image studio software (LI-COR). Data are expressed as fold-change normalized to the PSTAIR (Cdc28) loading control from three independent biological samples (±SEM, **p ≤ 0.005, ***p ≤ 0.0005 ****p < 0.0001). (**C**) Flow cytometry-based quantification of cell wall components in *Candida albicans*. Logarithmically growing cultures of SC5314 wild-type (wt), homozygous deletion (*gcn5*Δ/Δ) and restored (*gcn5*Δ/Δ::*GCN5*) Candida strains were washed and triple-stained to decorate cell wall components before quantification of β-D-glucan (FITC), chitin (BV421) and mannan (Texas Red) in suitable laser Channels. Data represents the mean fluorescence intensity (±SEM, *p < 0.05, **p < 0.01) from three independent experiments. (**D**) For qPCR quantification, RNA was extracted using the Trizol extraction method from *wt*, *gcn5*Δ/Δ and *gcn5*Δ/Δ::*GCN5* cultures as described in materials and methods. *FKS1*, *FKS2* and *FKS3* transcript levels were measured. Gene associated with Topoisomerase II (*PAT1*) mRNA was used as a normalization control and data represent mean relative expression to *PAT1* from three t <  six different experiments (±SEM, *p < 0.05, **p < 0.01, ***p<0.0005).

External stimuli trigger MAPK signaling pathways to transmit signals to dedicated downstream transcription factors that control morphogenesis^24,58,59^. To test how Gcn5 affects MAPK signaling, we immunoblotted for both activated and total levels of Mkc1, Cek1 and Hog1 (Fig. 2). Interestingly, activated Mkc1-P were 2-fold lower, whereas Cek1-P and Hog1-P showed 5-fold higher basal levels in *gcn5*∆/∆ mutant cells when compared to the total non-phosphorylated Mkc1, Cek1 and Hog1 controls (Fig. 2A,B), which remained unchanged in *gcn5*∆/∆ cells (Fig. 2A). No significant changes were observed in the restored *gcn5*∆/∆::*GCN5* strain (Fig. 2A,B). This differential activation of Mkc1, Cek1 and Hog1 in *gcn5*∆/∆ mutants implies a possible cross-talk between these MAPKs pathways that may, at least in part, account for altered cell wall phenotypes.

The fungicidal caspofungin (CSP) inhibits the Fks1-dependent synthesis and deposition of β-1, 3-D-glucan into the cell wall^14^. Notably, CSP treatment also elevates intracellular reactive oxygen species (ROS) and increases DNA damage. Vitamin C is an antioxidant that can quench ROS and protect cells from oxidative damage^39^. However, caspofungin hypersusceptibility was not due to elevated intracellular ROS, since *gcn5*∆/∆ cells actually produced less ROS upon caspofungin (Supplementary Fig. S2A,B).

### *GCN5* deletion alters cell surface components and *FKS* expression

We next analysed in more detail the effect of Gcn5 loss on cell wall architecture by quantifying all cell wall components (chitin, β1,3-glucan, branched β1,6-glucan and mannoproteins) using a FACS-based triple staining assay^60^. Both β-D-glucan and chitin levels were about 2-fold elevated in *gcn5*∆/∆ cells when compared to the wild type control or the restored strain. Outer mannoprotein levels were unchanged, indicating that *gcn5*∆/∆ causes specific cell wall defects rather than gross effects (Fig. 2C). We hypothesized that the increased β-D-glucan content in the *gcn5*∆/∆ mutant may be due to increased expression of glucan synthases such as *FKS*, which synthesises β-1, 3-D-glucan. To test this, we quantified mRNA expression levels of all three FKS genes, *FKS1*, *FKS2* and *FKS3*, encoded in the *C*. *albicans* genome. Unexpectedly, we found that expression of the CSP target Fks1 was significantly reduced upon removal of Gcn5, whereas both *FKS2* and *FKS3* were significantly upregulated (Fig. 2D). This implies a compensatory inverse regulation of *FKS* genes caused by the loss of Gcn5. Thus, we believe that the increased expression of *FKS1* upon CSP treatment in *wt* cells explains the CSP tolerance, which in turn is impaired when Gcn5 is ablated. In other words, the FKS2/3-triggered compensatory increase in glucan is not sufficient to restore CSP tolerance when *GCN5* is deleted. We cannot exclude a distinct glucan distribution or an aberrant integration of newly synthesized glucan into the functional cell wall architecture.

To test whether loss of Gcn5 alters cell membrane permeability, which may explain the altered drug sensitivity, we measured the kinetics of fluorescein diacetate (FDA) uptake, whose linear uptake is solely a function of lipid membrane permeability^61^ and thus independent of transporters (Supplementary Fig. S2C). Indeed, CSP increased FDA uptake in the wild type and restored strains, whereas basal FDA uptake in *gcn5*∆/∆ cells was already higher, indicating altered membrane lipid permeability that was not further increased by CSP (Supplementary Fig. S2C). To conclude, these results show that genetic ablation of *GCN5* alters the cell wall composition, FKS expression, and membrane lipid permeability.

### Transcriptional response of *wild type* and *gcn5*Δ/Δ cells upon caspofungin stress

**Figure 3:**
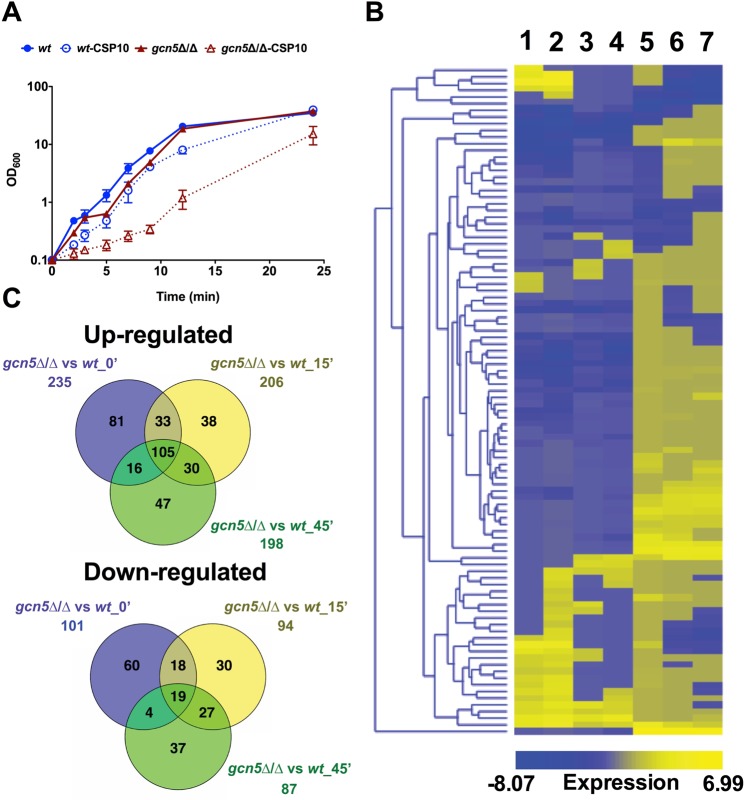
Transcriptional response of *wt* and *gcn5*Δ/Δ cells upon caspofungin stress. Total RNA was isolated from cultures of SC5314 wild-type (*wt*) and homozygous deletion strain (*gcn5*Δ/Δ) grown in the absence or presence of caspofungin and subjected to RNA-seq analysis. The bioinformatics pipeline for data analysis and hierarchial clustering is described in materials and methods. (**A**) Genetic removal of *GCN5* increases caspofungin susceptibility. Growth of indicated strains in YPD containing or lacking 10 ng/ml caspofungin (CSP10) at 30 °C. Data represent the means from three independent growth analyses (±SEM). Solid and dotted lines indicate untreated and caspofungin-treated cultures, respectively. (**B**) Heat map of hierarchical clustered and differentially expressed genes in *wt* and *gcn5*Δ/Δ cells in response to caspofungin treatment (CSP). Lanes 1 and 2 compare 15 min and 45 min CSP-treated *wt* cultures with untreated YPD-grown cells, respectively. Lanes 3 and 4 compare 15 min and 45 min CSP-treated *gcn5*Δ/Δ cultures with untreated YPD-grown cells, respectively. Lane 5 compares *gcn5*Δ/Δ with *wt* cells. Lanes 6 and 7 compare 15 min and 45 min CSP-treated *gcn5*Δ/Δ cultures with CSP-treated *wt* cells. Log-scaled expression values are colour-coded according to the legend on the bottom. (**C**) Venn diagrams depicting the overlap between up- (upper panel) and down-regulated (lower panel) genes after 0 min, 15 min and 45 min CSP-treatment of *gcn5*Δ/Δ cells compared to respective CSP-treated *wt* cells.

To dissect the Gcn5-dependent regulatory networks that may be underlying the observed phenotypes, we performed RNA-seq of *gcn5*∆/∆ cells and wild type in the presence and absence of CSP (Fig. 3). Growth curves and CSP sensitivity in liquid culture suggested a concentration of 10 ng/ml as appropriate, since this concentration exerted significant toxicity but still allowed for growth of all cells (Fig. 3A). The *gcn5*∆/∆ mutant showed sensitivity to 10 ng/ml CSP but retained more than 85% viability after 45 min but not at 60 min, where 30% cells were inviable (Fig. 3A; data not shown). Hence, we considered the time points 15- and 45-min post CSP treatment as optimal for RNA-seq. Differentially regulated genes were included when expression experienced a ≥ or ≤log_2_1.5-fold change, with an adjusted P-value cut-off ≤ 0.05 (Fig. 3, Supplementary Table S4).

We first compared transcript levels of YPD-grown *gcn5*∆/∆ to YPD-grown wild type cells and found 336 differentially regulated genes (Fig. 3B, lane 5). Out of these, 235 genes were induced, and 101 genes were repressed (Fig. 3C). The distribution of genes responding to *GCN5* deletion classified according to gene ontology (GO)-slim biological processes included major-enriched (cell adhesion, oxidation-reduction, transmembrane transport and pathogenesis) and minor-enriched (arginine biosynthesis, ergosterol and hydrogen sulphide biosynthesis, heme transport and zinc sequestration) as depicted in Supplementary Fig. S3A. Notably, genes implicated in pathogenesis were exclusively induced, except for the alpha-1,3-mannosyltransferase *MNN14*. Three-quarters of the differentially regulated genes were up-regulated in *gcn5*∆/∆ cells, implying that Gcn5 target genes are primarily negatively regulated. Together, our genome-wide analysis of *gcn5*∆/∆ cells suggests that *GCN5* deletion changes the landscape of several *C*. *albicans* transcriptional networks by regulating various target gene sets.

To determine whether alterations in the transcriptome potentially underlies the increased antifungal susceptibility of *gcn5*∆/∆ cells, we compared the effect of 15 and 45 min CSP treatment on the global transcriptomes of *gcn5*∆/∆ cells versus wild type cells (Fig. 3B, lanes 6 and 7). The Venn diagrams include the genes regulated at all time points from 0 min (untreated),15 min to 45 min CSP-treated in *gcn5*∆/∆ compared to wild type cells (Fig. 3C). We found that 15 min and 45 min CSP treatment showed approximately 70% and 50% overlap of induced (135 genes) and repressed (46 genes) genes, respectively (Fig. 3C). We also combined both 15 min and 45 min CSP-treated datasets and found that 154 genes were induced by CSP in both the *gcn5*∆/∆ and wild type cells. Some 81 genes were induced specifically in the absence of *GCN5*, while 115 genes were induced specifically upon CSP stress (Fig. 3C, upper panel). Surprisingly, given the differential sensitivities of the wild type and *gcn5*∆/∆ mutant to CSP, the CSP-mediated transcriptional response in wild type (Fig. 3B, lanes 1 and 2) and *gcn5*∆/∆ mutants (Fig. 3B, lanes 3 and 4) after 15 min and 45 min) revealed a striking overlap (Fig. 3B, lanes 1–4). The major biological processes differentially affected by the loss of Gcn5 included cell adhesion, oxidation-reduction, transmembrane transport, pathogenesis, lipid catabolism and iron homeostasis (Supplementary Fig. S3B). The inability of *gcn5*∆/∆ cells to fully induce the normal CSP response explains the increased antifungal susceptibility.

### Lack of *GCN5* affects oxidation-reduction, transport, pathogenesis and ion homeostasis

**Figure 4:**
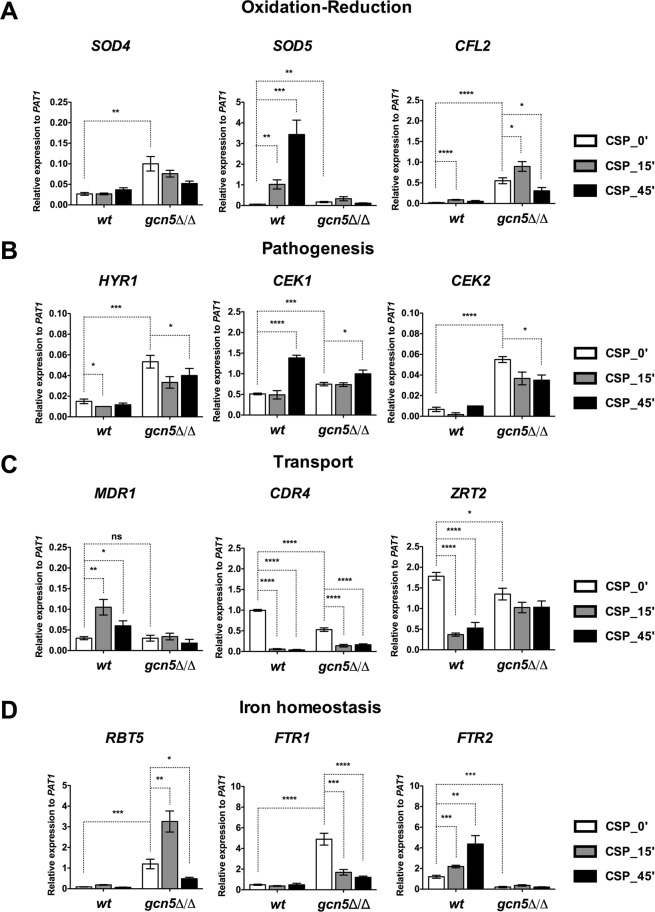
Genetic ablation of *GCN5* affects oxidation-reduction, transport, pathogenesis, iron homeostasis and adhesion genes. Quantitative real-time RT-PCR analysis of differentially expressed genes in YPD-grown *wt* and *gcn5*Δ/Δ cells measured in the presence or absence of caspofungin after the indicated time points. Data are shown as mean of relative expression to reference gene *PAT1* from three independent experiments (±SEM, *p < 0.05, **p < 0.01, ***p < 0.0005, ****p < 0.0001). Genes for validation were picked based on RNA-seq data and GO-term classification done by using online bioinformatics tool: Fungifun (https://elbe.hki-jena.de/fungifun/). (**A**) Genes implicated in oxido-reduction processes. (**B**) Genes implicated in fungal pathogenesis. (**C**) Genes implicated in transport and antifungal drug resistance. (**D**) Genes implicated in iron homeostasis.

To validate key predictions of the transcriptomics datasets, we employed quantitative real-time RT-PCR on hallmark genes known to be implicated in biological processes putatively affected by loss of Gcn5 (Supplementary Fig. S3A,B). Expression of at least three genes in each biological process was validated. The superoxide dismutase genes *SOD4* and *SOD5*, and the ferric reductase *CFL2* were previously implicated in oxidative stress resistance, as well as in the yeast-to-hyphae transition. In addition, *SOD5* has been linked to CSP-response^62–64^. Indeed, RT-qPCR analysis revealed approximately 4-fold, 3-fold and 29-fold increased *SOD4*, *SOD5* and *CFL2* mRNA levels, respectively, in *gcn5*∆/∆ cells when compared to the wild type (Fig. 4A). However, lack of *GCN5*, significantly elevated *CFL2* levels upon short-term treatment (CSP-15′), with a reduction at CSP-45′. As expected, CSP induced *SOD5* expression in wild type cells in a time-dependent manner (Fig. 4A), whereas, *gcn5*∆/∆ mutants were completely unable to induce *SOD5* upon CSP challenge.

Next, we checked the expression levels of *HYR1*, which encodes a GPI-anchored cell wall protein involved in hyphal regulation through the MAPK signaling genes *CEK1* and *CEK2*. These genes are required for the pathogenesis and cell wall-mediated invasion and pheromone responsive MAPK cascade, respectively^65,66^. Loss of *GCN5* induced approximately 4-fold, 1.5-fold and 8-fold of *HYR1*, *CEK1* and *CEK2* mRNAs, respectively. Prolonged CSP treatment slightly increased *CEK1* gene expression, contrary to the expression of *HYR1* and *CEK2* genes that were slightly repressed. 45 min CSP treatment induced *CEK1* expression by about ~3-fold (Fig. 4B). These results indicate that Gcn5 negatively regulates Cek1/Cek2 and Hyr1. Additional hyphal regulators include *EFG1*, *NRG1*, *TUP1*, *TEC1*, and hyphae-associated genes showing a predominant expression in filaments include *HWP1*, *ECE1* and *RBT5*^29,31,67^. Of note, these genes are also involved in biofilm formation and some are essential for fungal virulence^31,66–69^. Since, *GCN5* strongly affected hyphal formation (Fig. 1A,C), we tested whether additional hyphal regulators are also regulated by *GCN5*. Ablation of *GCN5* showed a ~5-fold and 2-fold basal upregulation of *HWP1* and *ECE1* transcripts, respectively. In contrast, *TEC1* mRNA was reduced ~2-fold when compared to wild type cells. Furthermore, expression of the repressor genes *NRG1* and *TUP1* were unaffected by the lack of Gcn5 (Supplementary Fig. S4).

Next, we quantified expression of the zinc permease Zrt2 and genes encoding multidrug resistance transporters such as the Mdr1 major facilitator and the ATP-binding cassette transporter Cdr4. In wild type cells, CSP induced *MDR1* expression, but significantly reduced expression of *CDR4* and *ZRT2*. Lack of *GCN5* rendered cells unable to drive CSP-responsive *MDR1* expression and reduced basal levels of *CDR4* and *ZRT2* (Fig. 4C). In addition, we quantified expression of iron homeostasis genes. The high-affinity iron permease genes *FTR1* and *FTR2* were differentially regulated upon loss of *GCN5* and CSP treatment, with 10-fold up and 6-fold down-regulated mRNAs in *gcn5*∆/∆ cells, respectively. Notably, expression of *FTR1* and *FTR2* encoding iron permeases was compromised upon CSP treatment in *gcn5*∆/∆ cells but induced in wild type in a time-dependent manner (Fig. 4D). Interestingly, *gcn5*∆/∆ cells showed 13-fold higher basal expression of the cell wall protein *RBT5*, with ~3-fold further upregulation upon CSP treatment (CSP-15′), whereas prolonged exposure significantly diminished *RBT5* expression (Fig. 4D). Taken together, removal of *GCN5* differentially regulates genes involved in oxido-reduction, ABC and MFS drug transporters, filamentation regulators, MAPK signaling modules, as well as iron / zinc homeostasis and heme acquisition, which requires Rbt5 and affects cell wall architecture by regulating its proteome including adhesins^70–74^.

### *GCN5* regulates cell adhesion through Efg1

**Figure 5:**
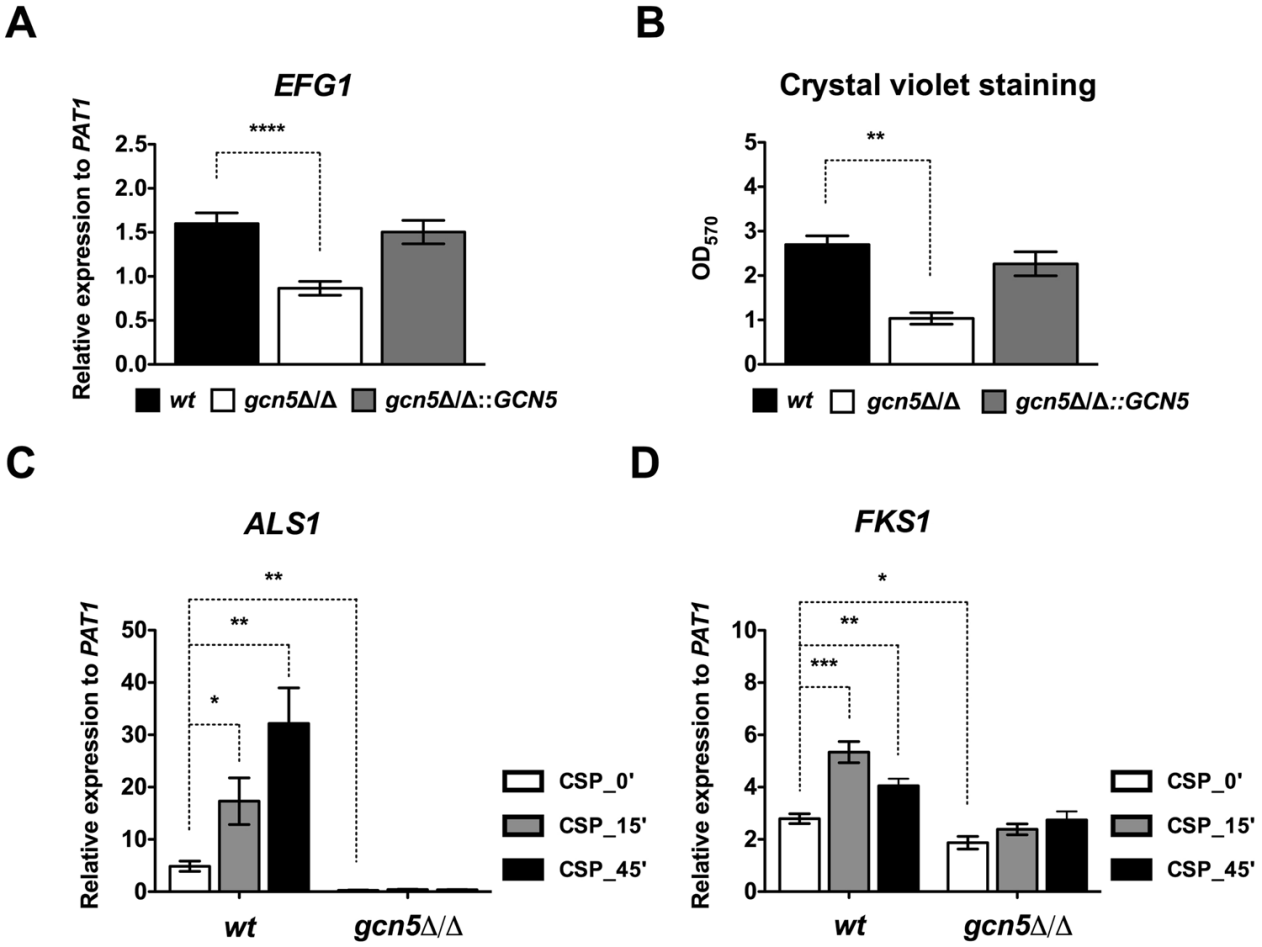
Loss of *GCN5* affects Efg1-mediated adhesion. Time course of qPCR-based quantification of mRNA levels in logarithmically growing cultures of SC5314 wild-type (wt), homozygous deletion (*gcn5*Δ/Δ) or restored (*gcn5*Δ/Δ::*GCN5*) Candida strains in the absence or presence of caspofungin. Data are shown as mean of relative expression to the reference gene *PAT1* from three independent experiments (±SEM, *p < 0.05, **p < 0.01, ***p < 0.0005, ****p < 0.0001). (**A**) *EFG1* expression in *wt*, *gcn5*Δ/Δ and *gcn5*Δ/Δ::*GCN5* cells. (**B**) Adherence on polystyrene-coated plates was measured via crystal violet staining of *wt*, *gcn5*Δ/Δ and *gcn5*Δ/Δ::*GCN5* cells, followed by the absorbance decay at 570 nm in the destaining solution containing 95% ethanol. Data are expressed as mean of crystal violet staining OD_600_ units from biological triplicates (±SEM, **p < 0.005). (**C**) Regulation of *ALS1* expression after caspofungin treatment in the absence of *GCN5* was measured as mentioned above. (**D**) Transcript levels of *FKS1* after caspofungin treatment.

The bHLH transcription factor Efg1 integrates signals through upstream cAMP-PKA pathways^31,35,75^ to control morphogenesis, CSP tolerance and adhesion^76^. Thus, we asked whether lack of *GCN5* affects Efg1 expression. We noticed a significant reduction in *EFG1* mRNA in *gcn5*∆/∆ cells when compared to wild type cells, which was not seen in restored strains (Fig. 5A), and confirmed the RNA-seq data (Fig. 3). Since Efg1 strongly impacted adhesion, we also quantified adherence properties by testing binding to polystyrene-coated plastic surfaces using the crystal violet readout. Indeed, cells lacking *GCN5* showed drastically attenuated adherence to polystyrene surfaces, which was not seen in the restored strain (Fig. 5B).

Efg1-mediated adhesion primarily engages the ALS agglutinin adhesion family^76^. Given that loss of *GCN5* increases susceptibility to CSP and decreases Efg1-mediated cell adhesion family, we tested the effect of CSP on Als1 expression (Fig. 5C). Wild type cells dramatically elevated Als1 levels upon CSP treatment, but the upregulation of *ALS1* was completely abolished in cells lacking Gcn5 (Fig. 5C). To reveal a potential role of other adhesins, we tested basal expression of all GPI-anchored *ALS* genes encoded in the Candida genome in wild type and *gcn5*∆/∆ mutants (Supplementary Fig. S5). Interestingly, like *ALS1*, *ALS2/3/4* were downregulated in *gcn5*∆/∆ cells, whereas *ALS5/6/7/9* mRNAs were significantly upregulated (Supplementary Fig. S5). Although the function of most *ALS* genes is unknown, the Gcn5-dependent dysregulation of well-established adhesins Als1 and Als3 explains the adhesion defects (Fig. 5C, Supplementary Fig. S5).

CSP inhibits β-1,3-D-glucan deposition by Fks1, whose overexpression confers resistance to CSP^77^. Wild type cells displayed significantly increased *FKS1* expression after 15 and 45 min CSP treatment. Conversely, *FKS1* mRNA was reduced in *gcn5*∆/∆ cells, and CSP treatment no longer increased *FKS1* mRNA levels (Fig. 5D), whereas both *FKS2* and *FKS3* were significantly upregulated (Fig. 2D). These data explain the CSP hyper-susceptibility and suggest a pivotal role of Gcn5 in regulating glucan deposition and cell wall function, including a coordinated action in concert with *EFG1*-mediated adhesion.

### Gcn5 is required for intracellular survival in BMDMs and essential for fungal virulence

**Figure 6:**
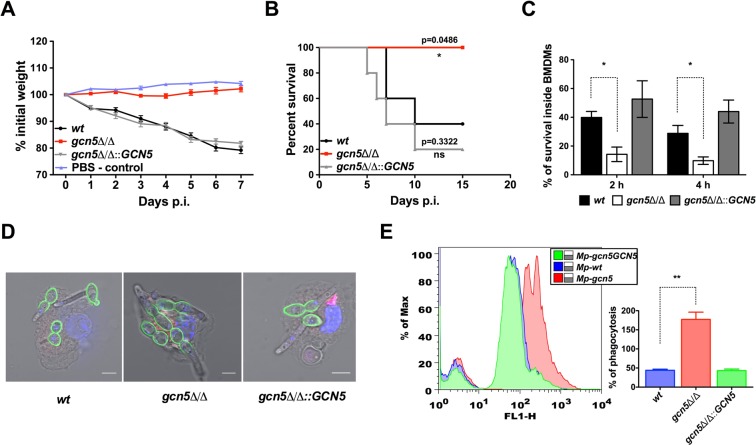
Lack of *GCN5* impairs survival inside BMDMs and leads to avirulence in mice. (**A**,**B**) Equal number of cells of logarithmically growing SC5314 wild-type (wt), homozygous deletion (*gcn5*Δ/Δ) and restored (*gcn5*Δ/Δ::*GCN5*) Candida strains were tail vein-injected (1 × 10^5^ cells/21 g mouse body weight). Mice were continuously monitored and weight loss was recorded as described in materials and methods. For survival experiments, mice were monitored for 15 days. A group five mice were used for each strain and log-rank test and the Kaplan Meier survival method was applied (*p < 0.05). (**C**) Logarithmically growing cells of SC5314 wild-type (wt), homozygous deletion (*gcn5*Δ/Δ) and restored (*gcn5*Δ/Δ::*GCN5*) Candida strains were used for infecting primary bone marrow-derived macrophages (BMDMs) at a multiplicity of infection (MOI) of 10:1 (fungi to macrophages). Fungal cells were harvested 2 h and 4 h post infection and viability quantified by cfu-counting on YPD plates as described in materials & methods to calculate the percentage of survival after 48 h incubation. Data represent the mean of three independent experiments (±SEM, *p < 0.05). (**D**) Confocal microscopy images of Alexafluor 488-labeled SC5314 wild-type (*wt*), homozygous deletion (*gcn5*Δ/Δ) or restored (*gcn5*Δ/Δ::*GCN5*) Candida strains infecting primary bone-marrow derived macrophages (BMDMs) at an MOI of 5:1 (fungi to macrophages). At 45 minutes post infection, cells were stained with Lyso Tracker Red DND-99 to visualize the phagolysosome, then fixed and further stained with DAPI to visualize the nucleus. Images shown are representative overlays of all four channels red, blue, grey and green to indicate staining of lysosomes, nucleus, Differential Interference Contrast (DIC) and *Candida albicans*, respectively. Scale bar = 5 µM. (**E**) Primary BMDMs were infected with Alexafluor 488-labeled *wt*, *gcn5*Δ/Δ and *gcn5*Δ/Δ::*GCN5* cells at an MOI of 2:1 (fungi to macrophages). At 45 minutes post infection, fluorescence of extracellular or adherent Candida was quenched by 0.4% trypan blue. After washing, intracellular phagocytosed Candida cells were quantified by flow cytometry. Data represent the mean of percent of phagocytosis (±SEM, **p < 0.005) from three independent assays. Representative overlay histogram to demonstrate the shift in fluorescence of *gcn5*Δ/Δ-infected BMDMs (red colour histogram). The mean fluorescence intensity of macrophage- internalized Candida were normalized to Candida alone or outside and represented as percent phagocytosis in a bar diagram shown next to the histogram.

Several phenotypes adopted by *gcn5*∆/∆ cells implied that Gcn5 may be involved in fungal virulence, as suggested by a previous report^56^. Thus, we investigated the role of *GCN5* in virulence *in vivo* using mouse models, and quantified the susceptibility of fungal cells to killing by innate immune cells. We used *a* murine Candia infection model and injected 1 × 10^5^ Candida cells through the lateral tail vein^78^. Mice infected with *gcn5*∆/∆ cells or mice receiving only PBS showed no weight loss. By contrast, mice receiving wild type or restored Candida strains lost approximately 20% of their weight over seven days (Fig. 6A). Remarkably, wild type and *GCN5* reconstituted cells efficiently killed animals, whereas *gcn5*∆/∆ cells were avirulent with all mice being able to clear the systemic infections (Fig. 6B).

To uncover a possible cellular mechanism, we challenged primary BMDMs with fungal strains in host-pathogen interaction experiments to quantify the killing capacity of macrophages. Macrophages are key components of the innate immune system as they engage in protecting the host by effective oxidative killing of *C*. *albicans*. To determine whether *GCN5* is required for intracellular survival of fungal cells after phagocytosis, we infected BMDMs with wild type, *gcn5*∆/∆ and restored strains using an MOI of 10:1 (Candida to BMDMs). Surviving fungal cells were quantified by CFU-counting after 2 h and 4 h of infection. Strikingly, *gcn5*∆/∆ were much more sensitive to killing by BMDMs when compared to wild type cells or *GCN5* reconstituted strains (Fig. 6C). Thus, loss of *GCN5* significantly reduced fungal viability inside BMDMs.

The impaired survival in macrophages prompted us to inspect the rate and degree of fungal phagocytosis using both fluorescence microscope and FACS-based detection of macrophage-internalized Alexafluor-488-labelled Candida cells (Fig. 6D,E). BMDMs showed enhanced engulfment and phagocytosis of fungal cells lacking *GCN5* when compared to wild type cells (Fig. 6D). These data are also consistent with the result showing abolished induction of *SOD5* in cells lacking Gcn5 (Fig. 4A), explaining the increased oxidative killing of fungal cells by BMDMs (Fig. 6E). Taken together, this works establishes that the fungal Gcn5 histone acetyltransferase is essential for fungal virulence and that pathogenicity is regulated through several independent mechanisms, including adhesion, antifungal susceptibility and cell wall architecture and a strikingly increased susceptibility to killing by host-derived oxidative stress. The data establish Gcn5 as a new potential target gene for antifungal drug discovery.

## Discussion

The prevalence and mortality of invasive fungal infections has been concerning. Importantly, the dramatic increases for fungal pathogens such as *Candida glabrata* in the past years, and the staggering increase in multidrug-resistant *Candida auris* infections, make fungal infections a global healthcare threat^1,79^. Therapeutic treatment options are limited to a small armory of antifungal drugs, some of which can lead to antifungal resistance or show restricted species-specificity. Hence, there is need to develop new and improved antifungal drugs, including an expansion of available chemical entities that could target new and understudied genes^80^. Compelling evidence indicates that enzymes that control chromatin modification could constitute a novel family of antifungal target genes, since they participate in numerous pathophysiological processes that regulate virulence traits^40^. Our work and data reported earlier^56^, are entirely consistent, and demonstrate that *C*. *albicans* Gcn5 is required for the hyphal formation and virulence in murine model of candidiasis. Here, we show that *C*. *albicans* Gcn5 controls morphogenesis and susceptibility to killing by phagocytes such as macrophages. Moreover, loss of Gcn5 is accompanied by Efg1-controled susceptibility to fungicidal caspofungin, as well as differentially altered MAPK signaling, which controls cell wall remodeling, adhesion and filamentation.

The Gcn5 histone lysyl acetyltransferaseis an intrinsic component of evolutionary conserved transcriptional co-activator complexes, such as SAGA, ADA and SLIK^46^, which regulate several fundamental processes^81–83^ in yeast^84–86^ and humans^45,46^. The knowledge about fungal Gcn5 primarily comes from studies in baker’s yeast^50,81,87^ and other fungal species^88,89^. Here, we use global transcriptomics and phenotypic profiling to identify regulatory networks and signaling pathways whose control engages Gcn5 in the human pathogen *C*. *albicans*. Although several phenotypes emerging upon deletion of *GCN5 in C*. *albicans* phenocopy those seen in yeast^84–86^, our data suggest that *Candida* spp Gcn5 may regulate additional networks required for virulence.

Gcn5-mediated regulation affects signaling pathways that are essential for controling morphogenetic changes such as filamentation as well as virulence. Hypersensitivities to caspofungin and other stress agents most likely arise as a consequence of altered cell wall composition and dysregulated glucan synthesis enzymes such as Fks1 and Fks2, Fks3. Interestingly, while we observed a strong repressive impact on *FKS1* in *gcn5*∆/∆ cells, compensatory adaptive mechanisms may drive *FKS2* and *FKS3* upregulation. However, we do not know whether this regulation is a direct or indirect mechanism activated upon loss of Gcn5. While *C*. *glabrata FKS1* and *FKS2* are functionally redundant, a recent report suggests that *C*. *albicans FKS2* and *FKS3* negatively regulate *FKS1*, which modulates echinocandin susceptibility^90,91^. These data suggest severe perturbations of cell wall homeostasis derived from loss of Gcn5 control cell wall architecture and function through dysregulated MAPK signaling^92^. The Hog1 and Cek1 pathways engage in molecular cross-talk^24^ and lack of Gcn5 indeed affects both pathways *C*. *albicans*.

Moreover, Gcn5 affects the synthesis of cell wall glucan and chitin through the Mkc1 cell integrity pathway^27^. Hence, our data suggest that Gcn5 differentially regulates MAPK signaling events, thereby altering cell surface composition. Indeed, the cell wall superoxide dismutase Sod5 is dramatically downregulated upon Gcn5 loss. Moreover, Gcn5 loss has a severe impact on adhesion as indicated by the dysregulation of the major Als1 adhesin, and the inability to induce Fks1 upon caspofungin stress. This dual regulation perhaps creates synthetic lethality under stress conditions, which could explain the attenuated virulence and dramatically impaired fitness in the host.

The prevailing notion that KATs in general, and Gcn5 specifically, functions as classical co-activators must be viewed with caution, since our RNA-seq data demonstrate that Gcn5, and possibly other Candida KATs, might also act as co-repressors, given the large number of about 235 genes upregulated after loss of Gcn5. For example, the elevated glucan and chitin contents imply a possible direct or indirect repressor function of Gcn5 by directly or indirectly modulating a positive transcriptional regulator required for cell wall homeostasis or adhesion. Further, the activation of both Cek1 and Hog1 MAPK pathways in the absence of stress may arise from constitutive cell wall alterations triggered by ectopic signaling. We speculate that Gcn5 alters chromatin remodelling in regulatory networks through its ability to acetylate histone H3 lysines (K14, K9, K18, K23, K27 and K36), as well as histone H4 and H2B^93^ when cells encounter stress or immune defense. However, it is tempting to further speculate that Gcn5 could also acetylate non-histone proteins, including signaling components of MAPK pathways or dedicated transcription factors to regulate their activity. Alternatively, Gcn5 could control the chromatin landscapes in promoters in virulence genes in cells that encounter active host immune defense^63,94^. Indeed, our initial characterization of Gcn5-associated proteins by native co-immunoprecipitation, and the identification of the Gcn5-dependent acetylome, suggests that Gcn5 interacts and acetylates several hundred target proteins, including numerous components of the ADA, SLIK, SAGA transcriptional regulatory complexes (Shivarathri *et al*., unpublished data). The RNA-seq data also reveal that Gcn5 modulates additional regulatory networks governing ergosterol lipid biosynthesis, oxido-reduction and cell adhesion, ABC and MFS transporter expression and antifungal resistance^95^, as well as iron homeostasis as in *C*. *glabrata*^96^. Interestingly, iron homeostasis is tightly linked to cell wall architecture, and Gcn5 regulates expression of Rbt5 that affects cell wall architecture by regulating adhesins and the entire surface proteome^70–74^. In addition, Gcn5 selectively controls hyphal regulator genes such as *EFG1*, *TEC1*, but also hyphal genes, including *HWP1* and *ECE1*. Of note, expression of *NRG1* and *TUP1* encoding hyphal repressors was not affected, implying that Gcn5 regulates hyphal elongation and filamentation primarily through Efg1-mediated control. In addition, deletion of *GCN5* considerably diminishes expression of ergosterol biosynthesis genes, including *ERG3*, *ERG250*, *ERG11*, *ERG13* and the binuclear Zn_2_-Cys_6_ transcription factor *UPC2*^97,98^ involved in the sterol uptake in yeast and Candida^97^. Hence, Upc2 may sense altered membrane lipid permeability, which is also linked to the overexpression of membrane transporters^99–102^. Indeed, *gcn5* mutants show changes in non-protein-mediated membrane lipid permeability and upregulation of the Cdr1 efflux pump^103^. This may explain, at least in part, *UPC2*-mediated alterations in antifungal drug susceptibilities emerging upon *GCN5* deletion^97^.

Moreover, ablation of Gcn5, strongly attenuates adhesion^104^, which requires Efg1 and determines immune cell interactions^16,105^. This is in line with previous reports showing that Efg1 modulates CSP tolerance through activating the major GPI-anchored cell surface adhesion protein Als1^76^. The Gcn5-mediated integration of filamentation signals also engages downstream transcription factors such as Efg1 and Tec1^29,106–108^ as both *EFG1* and *TEC1* expression is impaired in *gcn5* cells, thereby dysregulating Als1-mediated adhesion in *gcn5*∆/∆ cells facing CSP challenge. Of note, both *ALS1* and *ALS3* are also critically involved in fungal biofilm formation^109^, but a role for Gcn5 in biofilm control has not been tested.

Importantly, lack of Gcn5 significantly increases the susceptibility to killing by macrophages, because *gcn5*∆/∆ cells suffer from reduced fitness inside macrophages. The increased phagocytosis and or killing enables macrophages increased clearance of *gcn5*∆/∆ cells. This can explain for the most part the striking avirulence phenotype observed in a mouse model of systemic candidiasis, but additional unexplored mechanisms may also affect fitness of *gcn5*∆/∆ mutants. Of note, killing of cells lacking Gcn5 by dendritic cells is not affected, which may relate to their function as key antigen-presenting cells initiating adaptive immune responses^110^. The enhanced phagocytosis for *gcn5*∆/∆ may be due to increased exposure of cell surface 1,3-β-glucan, which triggers elevated ROS production by macrophages^111^. Similarly, yeast Gcn5 also modulates ROS-mediated cell death and oxidative stress^48^, although our results indicate that Gcn5-mediated ROS regulation in Candida excludes ROS produced upon caspofungin challenge^76^. Taken together, the impaired ROS scavenging by Sod5, the altered cell wall composition and adherence contribute to abrogating fungal virulence. However, the filamentation defects of *gcn5*∆/∆ cells might also cause reduced Sod5 induction, leading to increased susceptibility to killing. In fact, the reduced fitness may actually be a combinatorial effect, arising from defective Gcn5-mediated *SOD5* regulation (the major ROS-detoxifying SOD in Candida), impaired filamentation, along with the inability to escape from the phagosome. We speculate that Gcn5 may act in concert with Efg1 that integrates environmental and morphogenetic signals from upstream MAPK cascades, as well as protein kinase A (PKA) pathways^31,75,112^.

The *C*. *albicans* genome contains at least ten catalytic subunits^40,113^ with KAT activity, some of which may share redundant functions with Gcn5. For instance, other Candida KATs implicated in growth, morphogenesis and virulence include Ngg1, Esa1, as well as Hat1, Sas2 and Rtt109^36,38,39,114–116^. Of note, *GCN5*, *NGG1* and *ESA1* are required for filamentation and yeast-to-hyphal growth, whereas genetic ablation of *HAT1* and *SAS2* induces hyperfilamentation^37,115^ suggesting both positive and negative regulatory input by Candida KATs. Of note, the Gcn5-related acetyltransferase Ngs1 is required for sensing of the key filament inducer N-acetylglucosamine GlcNac. Ngs1 binds GlcNAc to activate its C-terminal acetyl transferase domain, driving promoter histone acetylation and transcription, including its promoter recruitment by the transcriptional regulator Rep1^117^.

KATs always act in concert with histone deacetylase in the regulation of both histone and non-histone targets, and like KATs, several *C albicans* KDACs such as Set3C, Rpd3, Rpd31, Hda1, Hos2, and Hst3 play essential roles in fungal virulence^34,40,118^. Therefore, KATs/KDACs are emerging drug targets^18,39,40,43,119^ amenable to antifungal drug discovery, as the current antifungal arsenal may become insufficient owing to intrinsic and pronounced drug resistance in *Candida glabrata*, and broad-spectrum antifungal resistance^14,120^ in emerging pathogens such as *C*. *auris*^12,121,122^. For example, the fungal KDAC inhibitor MGCD290, proved active in combination with fluconazole and echinocandins against drug-resistant Candida, as well as filamentous fungi^123,124^. Importantly, several Candida KATs target specific lysine residues on histones tails which are either absent or not modified by mammalian KAT orthologues. This suggests that fungal KAT inhibitors are unlikely to adversely affect mammalian KATs, making them especially suitable for drug discovery, and possibly useful for combination therapies^40,125^.

Taken together, our data suggest that Gcn5 holds new promises for therapeutic options to treat of invasive fungal infections, as Gcn5 controls several independent cellular and molecular pathways, all of which are critical for fungal fitness under immune surveillance. Interestingly, emerging evidence indicates that non-histone targets of KATs and perhaps KDACs could also play pivotal roles in fungal virulence and drug resistance^40,119^, opening yet another new window of opportunity for exploiting KATs/KDACS and their protein targets for antifungal drug discovery.

## Materials and Methods

### Ethics statement

All animal experiments were evaluated by the ethics committee of the Medical University of Vienna and approved by the Federal Ministry for Science and Research, Vienna, Austria (GZ: BMWF- 68.20n5/231-II/3b/2011) adhering to European legislation for animal experimentation.

### Strains and growth conditions

*Candida albicans* wild type and mutant strains were grown either in rich medium (YPD; 1% yeast extract, 2% peptone, and 2% dextrose) or in synthetically defined yeast nitrogen base (SC; 0.67% yeast nitrogen base and 2% dextrose) medium at 30 °C with shaking at 200 rpm. Logarithmic phase cells were obtained by growing overnight cultures in fresh yeast YPD/nitrogen base medium for 4 h at 30 °C. 200 µg/ml Nourseothricin was used as a selection marker for *C*. *albicans*. Bacterial strains were grown at 37 °C in LB medium containing either 120 µg g/ml ampicillin or 40 µg g/ml chloramphenicol. 2% agar was added to the plates. For bacterial transformations, SOC (add 20 mM glucose, 10 mM MgSO_4_, 10 mM KCl, 2.5 mM KH_2_PO_4_ to LB medium) media was used. The strains, plasmids, and primers used are listed in Supplementary Tables S1–S3.

### Fungalgene deletions and plasmid construction

Deletion of *GCN5* was performed by using the modified recyclable *NAT1* flipper method^36,57^. Briefly, upstream and downstream flanking regions of the *GCN5* gene were amplified using appropriate primers (Supplementary Table S1) covering approximately 50 bp of the homologous region to *NAT1* marker cassette (to replace the whole coding region of *GCN5*) and YEP352 plasmid which is containing ampicillin resistance marker and *E*. *coli* origin of replication. The FRT-FLP-NAT1-FRT cassette was amplified by using the plasmid pSFS3b. PCR amplified upstream, downstream fragments, *NAT1* marker cassette and YEP352 plasmids were cloned *in vivo* recombination either in *E*. *coli* EL350 cells or DH5α resulting in *gcn5∆* plasmid. Purified restriction digested *gnc5∆* plasmid was used to transform into *C*. *albicans* wild type cells. Two rounds of integration and excision generate homozygous *gcn5∆/∆* mutant. Transformation of *C*. *albicans* was done via electroporation exactly as described previously^57^.

### Growth and phenotypic characterization

For monitoring growth curves, overnight-grown Candida cultures were inoculated into YPD with or without caspofungin or into SC medium containing various carbon sources as indicated (initial OD_600_ of 0.1). Absorbance was recorded after various intervals and OD_600_ values were plotted versus time. Phenotypic characterization of *Candida albicans* mutants was done via serial-dilution spotting analysis on agar plates. Equal volumes (3 µl) of 10-fold serial dilutions of logarithmically growing *C*. *albicans* strains were spotted onto YPD plates containing different stress agents such as temperature stress (37 °C), serum (10%), caffeine (10 mM), SDS (0.05%), Congo Red (150 µg/ml), caspofungin (CSP, 100 and 150 ng/ml), hydrogen peroxide (H_2_O_2_, 4, 5 and 6 mM), Itraconazole (ITZ, 0.02 µg/ml), fluconazole (FLC, 2, 4 and 8 µg/ml), ketoconazole (KTZ, 0.05 µg/ml) and voriconazole (VCZ, 0.02 µg/ml) along with vitamin C (VitC, 25 mM) when indicated. Colony growth was scored after 48 h and compared to the YPD control plate. SC media plates containing different carbon sources such as glucose (2%), ethanol (2%), citric acid (2%), sodium acetate (2%), and glycerol (2%) were used to assess the utilization of alternative carbon sources.

### Filamentation assay

Logarithmically growing cells of SC5314 wild type (*wt*), homozygous deletion (*gcn5∆/∆*) and restored (*gcn5∆/∆*::*GCN5*) were plated on YPD plate supplemented with 10% fetal calf serum (FCS). Colony morphology was analysed after incubating plates for 3 days at 37 °C. Photographs were taken using a Discovery V12 Stereoscope equipped with an Axiocam MR5 camera (Zeiss). Scale bar corresponds to 1 mm.

### Calcoflour white staining and microscopy

To stain cell wall chitin with Calcoflour White (CFW; Fluorescent Brightener 28, Sigma), 1 ml aliquots of logarithmically growing cells were fixed in 4% *p*-formaldehyde for 1–2 hours, stained with CFW 1 mg/ml for 5 min. Differential Interference Contrast (DIC) and UV light images (UV) of the same cells are shown at 60x magnification. Scale bar = 5 µM. To inspect hyphal morphologies, 1 ml of logarithmically growing cells at 30 °C and 37 °C in YPD supplemented with 10% FCS were washed twice with PBS and fixed in 4% *p*-formaldehyde for 2 hours. Fixed cells were washed and images were taken with an LSM 700 Zeiss Confocal microscope at 60x magnification. Scale bar = 5 µM.

### ROS assay

Intracellular reactive oxygen species (ROS) was measured as described previously^37^ using the following modifications. Overnight grown Candida cultures were inoculated in SC medium containing 10% glucose at an initial OD_600_ of 0.3 and cultured at 30 °C for 1 h. The cultures contained 10% instead of 2% glucose to avoid CSP-induced flocculation^76^. Further, cultures were incubated with 20 mM dihydroethidium (DHE, Invitrogen) for 1 h, followed by caspofungin treatment (CSP, 150 ng/ml) for 150 min. Then, cells were washed once with water and samples were analysed by flow cytometry using FACS Calibur (BD Biosciences) at FL3-H channel. A minimum of 10,000 events were recorded for each sample. The data were analysed using Flowjo software (Flowjo LLC) and expressed mean relative fluorescence units from three independent different experiments.

### Flourescein diacetate (FDA) uptake assay

Candida strains were grown in the presence or absence of caspofungin (CSP, 50 ng/ml) for 4 h. About 5 × 10^6^ cells were resuspended and washed twice in 1 ml of FDA buffer before supplementing with 50 nm FDA. A 200 µl volume of cell mixture with or without FDA was added to an optical-bottom 96-well plate. The kinetics of FDA uptake was recorded every 5 min for 30 reads or until saturation was reached with simultaneous shaking of samples on the H1 Synergy plate reader with an excitation and emission wavelengths 485 and 535 nm, respectively. Data represent the mean fluorescence intensity over time. The slope rate was calculated using GraphPad Prism software.

### Western blot analysis

Logarithmically growing Candida cultures were washed once with ice-cold water and whole-cell extracts were prepared by trichloroacetic acid (TCA) method as described previously^126^. Extracts corresponding to 1 OD_600_ (1 × 10^7^ cells) were fractionated by 12% SDS-Page and blotted for proteins as indicated. Signals from the same whole cell extracts were detected using antibodies for total and active phosphorylated MAP kinases. The commercial antibodies recognized Mkc1 and Cek1 (p44/42 MAPK Erk1/2, Cell Signaling), and Hog1 (y-215, Santa Cruz), and phosphorylated Mkc1-P and Cek1-P (Phospho-p44/42 MAPK (Erk1/2), Cell Signaling) and Hog1-P (Phospho-p38, Cell Signaling). Reprobing with PSTAIR antibody (Sigma) recognizing Cdc28 served as a loading control. Protein bands on the nitrocellulose membrane were visualized using an Odysee® CLx scanner (Li-Cor®). Quantification of the protein band intensity was performed by using image studio software (LI-Cor®).

### Quantification of cell wall components by flow cytometry

Quantification of cell wall components by flow cytometry was performed as described previously^60^. Briefly, logarithmically growing cells of SC5314 wild type (*wt*), homozygous deletion (*gcn5∆/∆*) and restored (*gcn5∆/∆*::*GCN5*) strains were washed and stained with Concanavalin A-conjugated Texas Red, Dectin-1/Fc + 488 and CFW to decorate mannans, glucan and chitin, respectively. These triple-stained cells were measured in a BD Fortessa flow cytometer (BD biosciences) to quantify the amount of chitin, glucan and mannan using the BV421 (violet 405 nm, 50 mW power), FITC (blue 488 nm wavelength, 50 mW power) and Texas Red (red 640 nm wavelength, 40 mW power) lasers, respectively. A minimum of 10,000 events were recorded for each sample and the data were analysed using Flowjo software (Flowjo LLC). Unstained and single-stained samples served as controls and the data expressed as the mean fluorescence intensity from three independent experiments.

### Quantitative Real-time PCR (qPCR) analysis, RNA-seq analysis and bioinformatics

Total RNA isolation, cDNA synthesis and qPCR analysis was done as described previously^37^. The efficiency-corrected ΔΔCt method was used to quantify mRNA expression levels of a target gene transcript in comparison to a reference gene transcript^127^. The mRNA of the gene associated with Topoisomerase II (*PAT1*) was used as a reference gene^128^. GraphPad Prism software was used to perform statistical analyses of independent biological replicates as indicated.

For RNA-seq analysis, 5 μg DNase-treated total RNA was subjected to mRNA purification using Dynabeads mRNA purification kit (Invitrogen). Remaining rRNA contamination was checked in the bioanalyzer using Agilent RNA 6000 Pico kit (Agilent). Purified mRNA was subjected to fragmentation using the NEBNext® Magnesium RNA Fragmentation Module (New England Biolabs) and purified with RNeasy Plus Mini kit (Qiagen). Then, first-strand cDNA was synthesized with 50 ng/μl random hexamer primers using SuperScript ® III First-Strand Synthesis System for RT-PCR (Invitrogen). dNTPs were eliminated by purifying with MiniQuickSpin DNA Columns^129^ and subjected to second strand cDNA synthesis followed by clean-up with Minielute Reaction cleanup kit (Qiagen). cDNA concentration was measured using Quant-iT Picogreen dsDNA Reagents (Invitrogen) in a NanoDrop Fluorospectrophotometer ND-3300 (Thermo Fisher). cDNA samples were further processed for library preparation and sequenced on a HiSeq. 2500 (Illumina) at the Next Generation Sequencing Core Facility, Vienna Biocenter Core Facilities (VBCF, https://www.vbcf.ac.at/facilities/next-generation-sequencing). Three biological replicates for each time-points of wild type (*wt*) treated with caspofungin (CSP, 0, 15 and 45 min), as well as homozygous deletion (*gcn5*∆/∆) and two biological replicates for the remaining two time-points of *gcn5*∆/∆ (CSP, 15 min and 45 min) were sequenced. The RNA-seq data has been deposited in Gene Expression Omnibus (GEO) under the accession number GSE123412.

The bioinformatics analysis pipeline relied on the *C*. *albicans* genome Assembly 22 (http://www.candidagenome.org) to map sequence reads using TopHat, allowing for uniquely mapped reads^130^. HTSeq^131^ with union mode was used to assess the read counts using a reference annotation (C_albicans_SC5314_version_A22-s07-m01-r70; http://www.candidagenome.org). To identify the differentially expressed genes, read counts utilized DESeq2 R package^132^ with an adjusted P-value cut- off ≤ 0.05.

Heat map of hierarchical clustered and differentially expressed genes was generated using data mining tool Orange3^133^. Hierarchical clustering used the Euclidian distance and average cluster linking. The resulting dendrograms show the degree of similarity in gene expression. Venn diagrams were generated using Venny 2.1 (http://bioinfogp.cnb.csic.es/tools/venny)^134^. Gene ontology (GO) annotations were used the GO slim mapper tool (http://www.candidagenome.org/cgi-bin/GO/goTermMapper), and the online bioinformatics tool *Fungifun2* (https://elbe.hki-jena.de/fungifun/)^135^.

### Adherence assays

Adherence on polystyrene-coated plates was measured by crystal violet staining as described previously^136^ with minor modifications. Samples containing 2 × 10^7^ Candida cells in YPD were loaded into flat-bottomed 96-well microtiter plate (Corning) and incubated for 4 hours at 30 °C. The culture medium was aspirated and cells washed once with PBS to remove non-adherent Candida. Plate wells were allowed to dry at room temperature after 15 min methanol fixation and cells were stained for 5 min with 200 µl of 1% crystal violet (v/v). Then, cells were washed gently once with water, followed by the addition of 200 µl of 33% acetic acid for destaining, before absorbance was measured at 570 nm. Data are expressed as mean of absorbance of Candida strains from three independent technical as well as biological replicates.

### Phagocytosis assay

Flow cytometry-based phagocytosis assays were performed as described previously^36^. Wild type (*wt*), homozygous deletion (*gcn5∆/∆*) and restored (*gcn5∆/∆*::*GCN5*) cells were grown overnight to an OD_600_ of 1 and washed twice with PBS. Cells were stained with 10 mg/ml Alexa Fluor 488 (Life Technologies) in 100 mM HEPES buffer (pH 7.5) for 60 min at 30 °C with shaking in the dark. Stained Candida cells were washed thrice and resuspended in HEPES buffer, before infecting primary bone marrow-derived macrophages (BMDMs) at a multiplicity of infection (MOI) of 2:1. Phagocytosis was allowed and for 45 min at 37 °C and 5% CO_2_, before stopped by chilling samples on ice. Cells were fixed in 1% p-formaldehyde and stained with 0.4% trypan blue to quench the fluorescence of extracellular or adherent Candida. Control samples were kept on ice throughout the experiment. Intracellular phagocytosed Candida cells were quantified by flow cytometry analysis with FL1-H on a FACSCalibur (BD Biosciences). The data were analysed using Flowjo software (Flowjo LLC) and expressed as mean percentage of phagocytosis from three biological replicates.

Fluorescence microscopy-based phagocytosis assays were performed as described previously^137^, using minor modifications. BMDMs were infected with Alexafluor 488-labeled Candida strains at an MOI of 1:5 for 45 min, washed with PBS and further stained with 100 nM LysoTracker Red (DND-99) for 30 min prior to fixation. After 20 min in 3.7% formaldehyde fixation medium, cell were permeabilized for 15 min with 0.7% Triton X-100. DNA in permeabilized cells was stained with DAPI (49,6-diamidino-2-phenylindole) for 30 min. Microscopic slides were examined with a laser scanning microscope (LSM 700; Carl ZEISS). Images shown are representative overlays of all four channels red, blue, grey and green to indicate staining of lysosomes, nucleus, Differential Interference Contrast (DIC) and *Candida albicans*, respectively. Scale bar = 5 µM.

### Immune cells and mouse strains

Age-matched C57BL/6 wild type mice of 8–10 weeks were used for all experiments. Primary cultures of bone marrow-derived macrophages (BMDMs) and myeloid dendritic cells (mDCs) were isolated, and cultivated exactly as described before^138^. Survival of *C*. *albicans* in BMDMs and mDCs was quantified as described previously^63,139^ using an MOI of 10:1 (fungi to macrophages). Fungal cells were harvested 2 h and 4 h post infection and viability quantified by cfu-counting of samples on YPD. Survival was calculated as percentage of viable cfus after 48 h infection by comparing with uninfected Candida strains.

For virulence experiments, mouse infections were carried out as described previously^36,78^. Briefly, *C*. *albicans* strains were grown from frozen stocks overnight to an OD_600_ of around 1, washed twice and finally resuspended in PBS. For infection, 1 × 10^5^ *Candida* cells per 21 g body weight were injected into mice via the lateral tail vein. For survival experiments, mice were monitored for 15 days, including the recording of weight loss. A group of five mice were used for each strain. Statistical analysis was carried out using the GraphPad Prism software (Graphpad Software Inc.). Mouse survival curves used the log-rank (Mantle-Cox) test. For statistical analysis unpaired two-tailed Student’s t-test with 95% confidence intervals were used. P-values such as *p-value < 0.05; **p-value < 0.01; ***p-value < 0.005 were considered significant.

## Supplementary information

Supplementary Information

Supplementary Figures and Legends

Supplementary Tables S1-S3

Dataset 1

## Data Availability

The raw datasets and annotated gene expression analysis files have been deposited in the *Gene Expression Omnibus* (GEO; https://www.ncbi.nlm.nih.gov/geo/) database under the accession number GSE123412.
