# Supplementary material for: The Fungal Histone Acetyl Transferase Gcn5 Controls Virulence of the Human Pathogen Candida albicans through Multiple Pathways
Source: Sci Rep. 2019 Jul 1;9:9445. doi: 10.1038/s41598-019-45817-5 (PMC6603162; doi:10.1038/s41598-019-45817-5)
Supplement: Supplementary file 1 — Supplementary Information [file 41598_2019_45817_MOESM1_ESM.docx]

**Supplementary Information**

**The Fungal Histone Acetyl Transferase Gcn5 Controls Virulence of the Human Pathogen *Candida albicans* through Multiple Pathways**

Raju Shivarathri^1^, Michael Tscherner^1^, Florian Zwolanek^1^, Nitesh Kumar Singh^2^,

Neeraj Chauhan^3,4†^ and Karl Kuchler^1†^,

From The

^1^Medical University Vienna, Max F. Perutz Laboratories, Campus Vienna Biocenter, A-1030 Vienna, Austria

^2^Current Address: Qia ULB-Epigénétique du Cancer, Faculté Médecine

Route de Lennik 808, Bruxelles – Belgium

^3^Public Health Research Institute & ^4^Department of Microbiology, Biochemistry and Molecular Genetics, New Jersey Medical School, Rutgers

The State University of New Jersey, Newark, NJ 07103, USA

^†^ To whom correspondence can be addressed:

Karl Kuchler

Medical University Vienna, Max F. Perutz Laboratories

Department of Medical Biochemistry

Dr. Bohr-Gasse 9/2; A-1030 Vienna, Austria

Ph: +43-1-4277-61807; FAX: +43-1-4277-9618;

e-mail: [karl.kuchler@meduniwien.ac.at](mailto:Kkarl.Kuchlerkuchler@meduniwvien.ac.at)

and/or

Neeraj Chauhan

Public Health Research Institute - PHRI

Department of Microbiology, Biochemistry and Molecular Genetics

New Jersey Medical School

Rutgers, The State University of New Jersey

225 Warren Street, Newark, NJ 07103, USA

Ph: +1-973-854-3470 FAX: +1-973-854-3101

e-mail: [chauhan1@njms.rutgers.edu](mailto:chauhan1@njms.rutgers.edu)

**Supplementary Figure S1: Lack of the histone acetyltransferase Gcn5 alters susceptibility to caspofungin, azoles and cell wall stress agents.**

Equal volumes (3 µl) of 10-fold serial dilutions of SC5314 wild-type (*wt*), mutant (*GCN5/gcn5*Δ) and *gcn5*∆/∆ and restored (*gcn5*∆/∆::*GCN5)* Candida strains were spotted onto YPD plates containing different stress agents at the indicated concentrations. Colony growth was scored after 48 h and compared to the YPD control plate. **A.** temperature stress (37 °C), serum (10%), caffeine (10 mM), SDS (0.05%), Congo Red (150 µg/ml) and caspofungin (CSP, 100 and 150 ng/ml). **B.** Hydrogen peroxide (H_2_O_2_), Itraconazole (ITZ), fluconazole (FLC), ketoconazole (KTZ) and voriconazole (VCZ) were used at concentrations of 4, 5 and 6 mM, 0.02 µg/ml, 2, 4 and 8 µg/ml, 0.05 µg/ml and 0.02 µg/ml, respectively. **C.** Equal volumes (3 µl) of 10-fold serial dilutions of strains were spotted onto SC media plates containing different carbon sources such as glucose (2%), ethanol (2%), citric acid (2%), sodium acetate (2%), and glycerol (2%). Colony growth was scored after 48 h and compared to the control plate. **D.** Liquid growth of indicated strains at 30°C in SC medium containing various carbon sources as indicate. Data represent the mean (± SEM) from three independent experiments. **E.** Cell wall stress agents such as SDS, caffeine and caspofungin MIC distribution is shown*.* Data represent the mean (± SEM) from three independent experiments. Lines coloured in black, grey and red with dots corresponds to *wt*, *gcn5*Δ/Δ::*GCN5* and *gcn5*Δ/Δ cultures, respectively.

**Supplementary Figure S2: Caspofungin susceptibility in *gcn5* deletion is not due to increased ROS.**

**A.** Serial dilutions of indicated strains on YPD and YPD containing caspofungin (CSP, 100 and 150 ng/ml) alone or caspofungin supplemented with Vitamin C (VitC, 25 mM). Colony growth was scored after 48 h and compared to the YPD control plate. **B.** Reactive oxygen species measured inside indicated Candida strains in the presence or absence of caspofungin (CSP, 150 ng/ml) using dihydroethidium (DHE) staining and flow cytometry. Data represent mean relative fluorescence units from three different experiments (± SEM, ** = p<0.01, *** = p<0.0005, **** = p<0.0001). **C.** Indicated Candida strains were grown in the presence or absence of caspofungin (CSP, 50 ng/ml) for 4 h. 5x10^6^ cells were resuspended in 1 ml of FDA buffer with a final conc. of 50 nm FDA after two washes with FDA buffer. A 200 µl volume of cell mixture with or without FDA was added to an optical-bottom 96-well plate. The kinetics of fluorescence-based fluorescein diacetate (FDA) uptake was measured with continuous shaking every 5 min for 30 reads or until saturation was reached. Data represent the mean fluorescence intensity from three to four independent experiments (± SEM, ** = p<0.01, *** = p<0.0005). The rate constant (*K*) for YPD grown cultures was shown in the graph.

**Supplementary Figure S3: GO categories of genes regulated in *gcn5*∆/∆ cells upon caspofungin.**

GO annotations were used the Candida Genome Database (CGD) and GO slim mapper tool (<http://www.candidagenome.org/cgi-bin/GO/goTermMapper>) and the online bioinformatics tool *Fungifun2* (<https://elbe.hki-jena.de/fungifun/>). **A.** Distribution of genes in GO categories (biological process) in *gcn5*∆/∆ cells compared to *wt*. Genes up- or down-regulated with log_2_-fold values are indicated in pink and blue, respectively. **B.** Distribution of genes in GO categories (biological process) in the *wt* strain in response to caspofungin treatment compared untreated *wt*.

**Supplementary Figure S4: Expression of hyphal gene regulators.**

RNA was extracted from logarithmically growing SC5314 wild-type (*wt*), *gcn5*∆/∆ deletion and restored (*gcn5*∆/∆::*GCN5)* Candida strains using the trizol method. mRNA expression of *HWP1, TEC1, NRG1, ECE1* and *TUP1* genes was quantified by qPCR. Gene associated with Topoisomerase II (*PAT1*) mRNA was used as a normalization control. Data represent mean relative expression to *PAT1* from three to six different experiments (± SEM, ** = p<0.01, *** = p<0.0005, *** * = p<0.0001).

**Supplementary Figure S5: Disruption of *GCN5* differentially regulates the *ALS* adhesin gene family.**

RNA was extracted from logarithmically growing SC5314 wild-type (*wt*), *gcn5*∆/∆ deletion and restored (*gcn5*∆/∆::*GCN5)* Candida strains using the trizol method. qPCR-based quantification of mRNA levels of eight adhesin-encoding genes (*ALS1-7* and *ALS9*) was performed in indicated strains. Data are shown as mean of relative expression to the reference gene *PAT1* from three independent assays (± SEM, * = p<0.05, *** = p<0.0005).

**Supplementary Figure S6: Original digital images used to assemble Figure 2.**

Based on author guidelines, we provide the original uncropped immunoblots, which were used to assemble Figure 2. **A.** Odysee® settings were identical for all Western blots subjected to imaging. The white coloured boxes indicate the sections cropped from the original digital images of individual immunoblots, which were then used to compile Figure 2. Sections in Figure 2 obtained from independent immunoblots are separated by white spaces. **B.** Control immunoblots on whole cell extracts from fungal deletions as indicated to show antibody specificity. Coloured boxes indicate the lack of signals for a given MAPK in the corresponding fungal deletion strains.

**Supplementary Table S1:** *Candida albicans* strains used in this study.

**Supplementary Table S2:** Plasmids used in this study.

**Supplementary Table S3:** Primers used in this study.

**Dataset 1: Supplementary Table S4**

**Supplementary Table S4:** List of differentially expressed genes used for the generation of heatmap.
