# Supplementary material for: The Fungal Histone Acetyl Transferase Gcn5 Controls Virulence of the Human Pathogen Candida albicans through Multiple Pathways
Source: Sci Rep. 2019 Jul 1;9:9445. doi: 10.1038/s41598-019-45817-5 (PMC6603162; doi:10.1038/s41598-019-45817-5)
Supplement: Supplementary file 3 — Supplementary Tables S1-S3 [file 41598_2019_45817_MOESM3_ESM.docx]

**Supplementary Tables**

**The Fungal Histone Acetyl Transferase Gcn5 Controls Virulence of the Human Pathogen *Candida albicans* through Multiple Pathways**

Raju Shivarathri^1^, Michael Tscherner^1^, Florian Zwolanek^1^, Nitesh Kumar Singh^2^, Neeraj Chauhan^3,4†^ and Karl Kuchler^1†^,

From The

^1^Medical University Vienna, Max F. Perutz Laboratories, Campus Vienna Biocenter, A-1030 Vienna, Austria

^2^Current Address: Qia ULB-Epigénétique du Cancer, Faculté Médecine, Route de Lennik 808, Bruxelles – Belgium

^3^Public Health Research Institute & ^4^Department of Microbiology, Biochemistry and Molecular Genetics, New Jersey Medical School, Rutgers, The State University of New Jersey, Newark, NJ 07103, USA

^†^ To whom correspondence can be addressed:

Karl Kuchler

Medical University Vienna, Max F. Perutz Laboratories

Department of Medical Biochemistry

Dr. Bohr-Gasse 9/2; A-1030 Vienna, Austria

Ph: +43-1-4277-61807; FAX: +43-1-4277-9618;

e-mail: [karl.kuchler@meduniwien.ac.at](mailto:Kkarl.Kuchlerkuchler@meduniwvien.ac.at)

and/or

Neeraj Chauhan

Public Health Research Institute - PHRI

Department of Microbiology, Biochemistry and Molecular Genetics

New Jersey Medical School

Rutgers, The State University of New Jersey

225 Warren Street, Newark, NJ 07103, USA

Ph: +1-973-854-3470 FAX: +1-973-854-3101

e-mail: [chauhan1@njms.rutgers.edu](mailto:chauhan1@njms.rutgers.edu)

**Supplementary Table S1. *Candida albicans* strains used in this study**

| **Description** | **Name** | **Parent** | **Genotype** | **Reference** | **Figures** |
| --- | --- | --- | --- | --- | --- |
| Wild type (wt) | SC5314 | Clinical isolate |  | ^1^ | Figures 1, 2, 3, 4, 5, 6 |
| *GCN5/gcn5*∆ | CA-MT238 | SC5314 | *GCN5/gcn5*Δ::*NAT1*-FLP | This study |  |
| *GCN5/gcn5*∆ | CA-MT240 | CA-MT238 | *GCN5/gcn5*Δ::FRT | This study |  |
| *gcn5*∆/∆ | CA-MT243 | CA-MT240 | *gcn5*Δ*/gcn5*Δ::*N*AT1-FLP | This study |  |
| *gcn5*∆/∆ | CA-MT569 | CA-MT243 | *gcn5*Δ*/gcn5*Δ::FRT | This study | Figures 1, 2, 3, 4, 5, 6 |
| *gcn5*∆/∆::*GCN5* | CA-MT570 | CA-MT569 | *gcn5*Δ*/gcn5*Δ::*GCN5*::*NAT1*-FLP | This study |  |
| *gcn5*∆/∆::*GCN5* | CA-MT571 | CA-MT570 | *gcn5*Δ*/gcn5*Δ::*GCN5*::FRT | This study | Figures 1, 2, 5, 6 |
| *CEK1/cek1*∆ | CA-RS2 | SC5314 | *CEK1/cek1*Δ::*NAT1*-FLP | This study |  |
| *CEK1/cek1*∆ | CA-RS33 | CA-RS2 | *CEK1/cek1*Δ::FRT | This study |  |
| *cek1*∆*/cek1*∆ | CA-RS52 | CA-RS33 | *cek1*∆*/cek1*∆::*NAT1*-FLP | This study |  |
| *cek1*∆*/cek1*∆ | CA-RS61 | CA-RS52 | *cek1*∆*/cek1*∆::FRT | This study |  |
| *gcn5*∆/∆::*CEK1/cek1*∆ | CA-RS3 | CA-MT243 | *gcn5*Δ*/gcn5*Δ::*CEK1/cek1*Δ::*NAT1*-FLP | This study |  |
| *gcn5*∆/∆::*CEK1/cek1*∆ | CA-RS40 | CA-RS3 | *gcn5*Δ*/gcn5*Δ::*CEK1/cek1*Δ::FRT | This study |  |
| *gcn5*∆/∆::*cek1*∆*/cek1*∆ | CA-RS79 | CA-RS40 | *gcn5*Δ*/gcn5*Δ::*cek1*∆*/cek1*∆::*NAT1*-FLP | This study |  |
| *gcn5*∆/∆::*cek1*∆*/cek1*∆ | CA-RS94 | CA-RS79 | *gcn5*Δ*/gcn5*Δ::*cek1*∆*/cek1*∆::FRT | This study |  |
| *HOG1/hog1*∆ | CA-RS7 | SC5314 | *HOG1/hog1*Δ::*NAT1*-FLP | This study |  |
| *HOG1/hog1*∆ | CA-RS13 | CA-RS7 | *HOG1/hog1*Δ::FRT | This study |  |
| *hog1*∆*/hog1*∆ | CA-RS49 | CA-RS13 | *hog1*∆*/hog1*∆::*NAT1*-FLP | This study |  |
| *hog1*∆*/hog1*∆ | CA-RS63 | CA-RS49 | *hog1*∆*/hog1*∆::FRT | This study |  |
| *gcn5*∆/∆::*HOG1/hog1*∆ | CA-RS9 | CA-MT243 | *gcn5*Δ*/gcn5*Δ::*HOG1/hog1*Δ::*NAT1*-FLP | This study |  |
| *gcn5*∆/∆::*HOG1/hog1*∆ | CA-RS15 | CA-RS9 | *gcn5*Δ*/gcn5*Δ::*HOG1/hog1*Δ::FRT | This study |  |
| *gcn5*∆/∆::*hog1*∆*/hog1*∆ | CA-RS51 | CA-RS15 | *gcn5*Δ*/gcn5*Δ::*hog1*∆*/hog1*∆::*NAT1*-FLP | This study |  |
| *gcn5*∆/∆::*hog1*∆*/hog1*∆ | CA-RS68 | CA-RS51 | *gcn5*Δ*/gcn5*Δ::*hog1*∆*/hog1*∆::FRT | This study |  |
| *mkc1*∆*/mkc1*∆ | CA-RS95 | SC5314 | *mkc1*∆*/mkc1*∆::*NAT1*-FLP | This study |  |
| *mkc1*∆*/mkc1*∆ | CA-RS97 | CA-RS95 | *mkc1*∆*/mkc1*∆::FRT | This study |  |
| *gcn5*∆/∆::*mkc1*∆*/mkc1*∆ | CA-RS96 | CA-MT243 | *gcn5*Δ*/gcn5*Δ::*mkc1*∆*/mkc1*∆::*NAT1*-HIS/FLP | This study |  |
| *gcn5*∆/∆::*mkc1*∆*/mkc1*∆ | CA-RS102 | CA-RS96 | *gcn5*Δ*/gcn5*Δ::*mkc1*∆*/mkc1*∆::FRT | This study |  |

**Supplementary Table S2. Plasmids used in this study**

| **Plasmids** | **Parent** | **Relevant inserts and cloning sites** | **Reference** |
| --- | --- | --- | --- |
| pSFS2a |  |  | ^2^ |
| pSFS3b | pSFS2a | FRT-FLP-NAT1-FRT-BglII | ^3^ |
| pSFS3b-*GCN5 5’utr/3utr’* | pSFS3b | 5' *GCN5*-FRT-FLP-*NAT1*-FRT-3' *GCN5* | This study |
| pSFS3b-*GCN5 int* | pSFS3b-*GCN5 5’utr/3utr’* | 5' *GCN5, GCN5 CDS-*3' *GCN5*-FRT-FLP-*NAT1*-FRT-3' *GCN5* | This study |
| pSFS3b-*CEK1 5’utr/3utr’* | pSFS3b | 5' *CEK1*-FRT-FLP-*NAT1*-FRT-3' *CEK1* | This study |
| pSFS3b-*HOG1 5’utr/3utr’* | pSFS3b | 5' *HOG1*-FRT-FLP-*NAT1*-FRT-3' *HOG1* | This study |
| pADH99 |  | HIS/FLP CAS9 expression plasmid | ^4^ |
| pADH110 |  | Universal plasmid template for Fragment A for cloning-free stitching of gRNA expression cassette | ^4^ |
| pADH119 |  | Plasmid template for Fragment B for cloning-free stitching of gRNA expression cassette | ^4^ |
| pADH147 |  | HIS/FLP plasmid template for Fragment B for cloning-free stitching of gRNA expression cassette | ^4^ |

**Supplementary Table S3. Primers used in this study**

Gene deletion oligonucleotides in 5’-3’ direction used in the *SAT1*-flipping method and CRISPR-Cas9 method

| **Name** | **Sequence (5'→3')** | **Reference** |
| --- | --- | --- |
| SATflipp_oe_fwd | GGGCGAATTGGGTACCGG | ^2^ |
| SATflipp_oe_rev | CCATGATTACGCCAAGCGC | ^2^ |
| SATflipp_5C | TTTGGAACTAACGATGCATACGAC | ^2^ |
| SATflipp_3C | CCTAACATATGTGAAGTGTGAAGGG | ^2^ |
| heukan3 | CATCATCTGCCCAGATGCGAAG | ^2^ |
| 5C_GCN5 | CGTCTAAGACACAAGGCTAGC | This study |
| 55_GCN5_PvuIKpnI | atgcgatcggtaCCATTGTCCAGGTGGTTG | This study |
| 53_GCN5_ApaI | atcgggcccTACGAATTGTTATTGATCGG | This study |
| 35_GCN5_BglII | atcagatCTGTTTTGACGAATATATGATATG | This study |
| 33_GCN5_NotI | atcgcggccgcGAATCAGGATGATACAATGG | This study |
| 3C_GCN5 | CATGTTTGTGGTCTCTTGAGG | This study |
| GCN5 CDS_BamHI_fwd | atcggatccATGGTTGACAGAAAAAGAAC | This study |
| GCN5 CDS_BamHI_rev | atcggatcCTATACAAAACTACAGTCTTTC | This study |
| RT5_GCN5 | TGGATTAGCAGAGGCAGG | This study |
| RT3_GCN5 | GGGTGGTTCTGAATTTCACTG | This study |
| 5C_CEK1 | CTCCAATACACACACAATCCCAC | This study |
| 3C_CEK1 | GTCTGATATTAATTTGGCTTGCTG | This study |
| 55_CEK1_KpnI | actgggtaccGTGTTTATATTCAGACCAACTC | This study |
| 53CEK1_ApaI | actggggcccGTAGTAGCGATTTCTATTGATG | This study |
| 35_CEK1_BglII | actgagatctGAGGTCAAGCTAAGTTAAGC | This study |
| 33_CEK1_NotI | actggcggccgcGAGATTTAATAACACCGCCAC | This study |
| CEK1_LOG_fwd | GATTTACATCGAGTCATTAGAACTC | This study |
| CEK1_LOG_rev | CATCTTCAACAGTAATTCTTTTAGC | This study |
| 5C_HOG1 | CACCTCAGCTAGTAACACTACTG | This study |
| 55_HOG1 | agctatgaccatgattacgaattcgagctcggtacccgggTCAGTTTCGATAATGACATC | This study |
| 53_HOG1 | tctctagaaagtataggaacttcctcgagggggggcccggTCTTATATGCTTTATCTTTG | This study |
| 35_HOG1 | atccactagttctagagcggccgccaccgcggtggagctcCTTCAAAAATACAAGCTAGC | This study |
| 33_HOG1 | cgacgttgtaaaacgacggccagtgccaagcttgcatgcCAGAAGACAATCTTTTGAAC | This study |
| 3C_HOG1 | CTAATTTATCAAAACAATACCCTC | This study |
| HOG1_LOG_fwd | CTTCAAGATCCACAAATGACGG | This study |
| HOG1_LOG_rev | CATCATAACTCTCCAAGTATCCAC | This study |
| HOG1 CDS_rev | agttcctattctctagaaagtataggaacttcctcgagggggggcccggTTAAGCTCCGTTGGCGGAATC | This study |
| MKC1_gRNA1 | cgtaaactatttttaatttgTTATCCCAGACCACAAGAGTgttttagagctagaaatagc | This study |
| MKC1_gRNA2 | cgtaaactatttttaatttgTCAGACCCCTTACAATAACTgttttagagctagaaatagc | This study |
| MKC1_gRNA3 | cgtaaactatttttaatttgGAACTGTAATTCTCTCACGTgttttagagctagaaatagc | This study |
| MKC1_repair_fwd | TTTTTAAACTTTCTCTTGAACAGCAGTTTTATAAAGAACCAATTTCCATATTATCCCGGGGTATGTTACTCTCGAATTAGTCTATCCATTAGTGGTGGTT | This study |
| MKC1_repair_rev | AACCACCACTAATGGATAGACTAATTCGAGAGTAACATACCCCGGGATAATATGGAAATTGGTTCTTTATAAAACTGCTGTTCAAGAGAAAGTTTAAAAA | This study |
| AHIO1096 | GACGGCACGGCCACGCGTTTAAACCGCC | ^4^ |
| AHO1097 | CCCGCCAGGCGCTGGGGTTTAAACACCG | ^4^ |
| AHO1098 | caaattaaaaatagtttacgcaag | ^4^ |
| AHO1236 | TAAAGCTGCCACAAGAGGTATTTC | ^4^ |
| AHO1237 | aggtgatgctgaagctattgaag | ^4^ |
| **Primers used for RT-qPCR** | | |
| RT5_TUP1 | GATGGCGATAGGTTGGTTTCAG | ^5^ |
| RT3_TUP1 | ACAAAGTCAAGGAACACTGGGAG | ^5^ |
| RT5_TEC1 | TGAGCAACAACAACAACAACCAC | ^5^ |
| RT3_TEC1 | CTGGGTTGTTGTCATAGTGGCC | ^5^ |
| RT5_ECE1 | TGCCATTTGTTGTCAGAGCTG | ^6^ |
| RT3_ECE1 | TAGCTTGTTGAACAGTTTCCAGG | ^6^ |
| RT5_EFG1 | CATCACAACCAGGTTCTACAACCAAT | ^6^ |
| RT3_EFG1 | CTACTATTAGCAGCACCACCC | ^6^ |
| RT5_HWP1 | GCTGGTTCAGAATCATCCATGC | ^6^ |
| RT3_HWP1 | AAGGTTCAGTGGCAGGAGCTG | ^6^ |
| RT5_NRG1 | GGTTGCACGTTGTCGAAACC | ^6^ |
| RT3_NRG1 | TGTTGCTGCTGCTGCTTGG | ^6^ |
| RT5_RBT5 | ACAGAATCTACGACCAATTGCCAG | This study |
| RT3_RBT5 | CAGCAGATGAACAAATGGAAGTG | This study |
| RT5_ALS1_2 | GCAAGTACAATGTCTGATTCAC | This study |
| RT3_ALS1_2 | GATGTCACTGAAGATGATAATGGG | This study |
| RT5_ALS2 | AGAGAACCACCAAATTACACAG | This study |
| RT3_ALS2 | ACCAATACTCGGTTGTAGTGAC | This study |
| RT5_ALS3 | CTCGTCCTCATTACACCAACC | This study |
| RT3_ALS3 | GAAACAGAAACCCAAGAACAAC | This study |
| RT5_ALS4 | CAATAGTGTCATTATCCGAGTCC | This study |
| RT3_ALS4 | TAGGTAAGTGGGTAACTGTAGAG | This study |
| RT5_ALS5 | TGACTACTTCCAGATTTATGCC | This study |
| RT3_ALS5 | CCATAACTAGTGGAGAATCCCA | This study |
| RT5_ALS6 | GACTCCACAATCATCTAGTAGC | This study |
| RT3_ALS6 | CACACCGGATGCATATCCA | This study |
| RT5_ALS7 | GAAGAGAACTAGCGTTTGGT | This study |
| RT3_ALS7 | CTGGTAACCCATGAAACAAGG | This study |
| RT5_ALS9 | AACTGGAAGCTCCAAGTCTC | This study |
| RT3_ALS9 | GAAACTGAAACTGCTGGATTTGG | This study |
| RT5_FKS1 | TGATACTGGTAATCATAGACCAAAAA | This study |
| RT3_FKS1 | AACTCTGAATGGATTTGTAGAATAAGG | This study |
| RT5_FKS2 | ACTTGCTAGCAGTCGCCAAT | This study |
| RT3_FKS2 | ACCACCATGAGCGGTTAGAC | This study |
| RT5_FKS3 | ACCTCAATATTCAGCTTGGTGCCC | This study |
| RT3_FKS3 | GGACAACTCATTCGACTTGACCGT | This study |
| RT5_CFL2 | GCCAAGAAATCAAAGAACCAGTC | This study |
| RT3_CFL2 | TCTAACTCTTCCGTAAACCAACTC | This study |
| RT5_CEK1 | GTTGAGTGGTAGACCTTTATTCCC | This study |
| RT3_CEK1 | TGGCAAATAATTCACTGAACGG | This study |
| RT5_CEK2 | GGAACACCAACCGATGAAGAC | This study |
| RT3_CEK2 | GCTAATGGGTTCACTTGGTCTC | This study |
| RT5_HYR1 | GGAAATGGTTCTGAAGCTGG | This study |
| RT3_HYR1 | AGTGGCAGTAGCATCAGTGTG | This study |
| RT5_MDR1 | TCATTGCTTCAGTGTTCCCA | This study |
| RT3_MDR1 | CTGGAATAGCAATCATAACAAGGG | This study |
| RT5_CDR4 | CTGGAACTAGACTTGATGCTGGA | This study |
| RT3_CDR4 | ACAAATACTGCTCCCAATAAGGTG | This study |
| RT5_ZRT2 | TGATGCCAATCCATATCCATCTC | This study |
| RT3_ZRT2 | CCAAAGCAAGACCTATGAATACTG | This study |
| RT5_SOD5_464s | CAATGGTACCAGATTGAACTGTGC | ^7^ |
| RT3_SOD5_582as | AAGAAGTGTTGACTGCACTTTGAG | ^7^ |
| RT5_SOD4_417s | GACAGTAAAGCTTACATTGGTGGG | ^7^ |
| RT3_SOD4_529as | GCACTTGCAGTATCGTCACC | ^7^ |
| RT5_2 PAT1 | CAGCAACTGATTTATCGGAATGG | ^7^ |
| RT3_2 PAT1 | ACATCTTCAGGGTTAGGTGG | ^7^ |

**Supplementary references**

1 Gillum, A. M., Tsay, E. Y. & Kirsch, D. R. Isolation of the *Candida albicans* gene for orotidine-5'-phosphate decarboxylase by complementation of *S. cerevisiae* ura3 and E. coli pyrF mutations. *Mol Gen Genet* **198**, 179-182 (1984).

2 Reuss, O., Vik, A., Kolter, R. & Morschhauser, J. The *SAT1* flipper, an optimized tool for gene disruption in *Candida albicans*. *Gene* **341**, 119-127, doi:10.1016/j.gene.2004.06.021 (2004).

3 Tscherner, M., Stappler, E., Hnisz, D. & Kuchler, K. The histone acetyltransferase Hat1 facilitates DNA damage repair and morphogenesis in *Candida albicans*. *Molecular microbiology* **86**, 1197-1214, doi:10.1111/mmi.12051 (2012).

4 Nguyen, N., Quail, M. M. F. & Hernday, A. D. An Efficient, Rapid, and Recyclable System for CRISPR-Mediated Genome Editing in *Candida albicans*. *mSphere* **2**, doi:10.1128/mSphereDirect.00149-17 (2017).

5 Hnisz, D. *et al.* A histone deacetylase adjusts transcription kinetics at coding sequences during *Candida albicans* morphogenesis. *PLoS Genet* **8**, e1003118, doi:10.1371/journal.pgen.1003118 (2012).

6 Jenull, S. *et al.* The *Candida albicans* HIR histone chaperone regulates the yeast-to-hyphae transition by controlling the sensitivity to morphogenesis signals. *Sci Rep* **7**, 8308, doi:10.1038/s41598-017-08239-9 (2017).

7 Tscherner, M. *et al.* The *Candida albicans* Histone Acetyltransferase Hat1 Regulates Stress Resistance and Virulence via Distinct Chromatin Assembly Pathways. *PLOS Pathogens* **11**, doi:10.1371/journal.ppat.1005218 (2015).
